# Nanotechnology and Agricultural Sustainability: A Review

**DOI:** 10.3390/nano15231755

**Published:** 2025-11-22

**Authors:** Siqi Zeng, Noman Shakoor, Yukui Rui

**Affiliations:** 1 State Key Laboratory of Nutrient Use and Management, China Agricultural University, Beijing 100193, China; zengskey@163.com; 2 College of Life Sciences and Oceanography, Shenzhen University, Shenzhen 518060, China; nomanshakoor1993@yahoo.com; 3 Hebei Wuqiang County Professor Workstation and Science and Technology Small Courtyard, China Agricultural University, Hengshui 053300, China

**Keywords:** nanotechnology, precision agriculture, sustainable approaches, applied research

## Abstract

Nanotechnology plays a crucial role in promoting precision agriculture and environmental management. This review integrates the latest advances in nanotechnology in the fields of pollution detection, agrochemicals, and stress resistance, and quantifies the significant enhancements brought by nanomaterials (NMs). NMs used in biosensors enable highly sensitive, low detection limit, and highly accurate detection of environmental pollution, plant growth status, and soil conditions, while achieving precise drug delivery and reducing environmental pollution. Furthermore, NMs can be combined with agrochemicals or directly act on plants to promote growth, reduce pests and diseases, and enhance stress resistance by altering plant physiological processes and microbial functions. This review focuses on the application value of nanotechnology in detection, smart chemicals, and stress resistance, and analyzes current challenges and risks in technology, biosafety, regulatory challenges, and scalability. Finally, it points out future directions for utilizing nanotechnology to advance smart agriculture, precision agriculture, and green bio-industrialization.

## 1. Introduction

By 2050, the United Nations estimates that the world’s population will reach 9.8 billion [1]. To meet the increasing food demand driven by population growth, global food production needs to increase by 70% [2]. However, traditional agricultural methods are struggling to meet this demand while simultaneously placing significant pressure on the environment. Conventional agricultural practices heavily rely on synthetic fertilizers and pesticides. However, a substantial portion of these inputs is not absorbed by crops; instead, they are lost to the environment through volatilization into the atmosphere, runoff into water bodies, and leaching into groundwater [3]. This leads to a series of negative impacts, including water eutrophication, greenhouse gas emissions, and soil nutrient imbalance [4,5]. Furthermore, the use of plastic mulching films has resulted in their persistent accumulation in soils, causing irreversible microplastic pollution [6]. Consequently, future agricultural development must increasingly rely on technological breakthroughs to overcome these limitations and enhance agricultural productivity.

As an emerging approach, nanotechnology has been widely applied in fields such as energy, manufacturing, medical diagnostics, pharmaceuticals, biology, and the environment due to its characteristics of high specific surface area, controlled release, stability, targeting, and signal transduction [7]. The core of nanotechnology comprises nanomaterials (NMs), which typically range in size from 1 to 100 nm. Based on their dimensional size range, NMs can be categorized into zero-dimensional (0D), one-dimensional (1D), two-dimensional (2D), and three-dimensional (3D) materials [8]. The ultrafine size of NMs enables them to penetrate cell membranes, induce biochemical reactions in living cells, and influence metabolic processes, growth, and development [9]. Numerous studies have shown that NMs positively affect the growth and development of crops [10,11]. For example, SiO_2_ NMs can promote the germination and growth of rice seeds [12], TiO_2_ can promote the growth and development of wheat and increase the nutrient content [13], MoS_2_ NMs can increase soybean yield and nitrogen fixation efficiency [14]. Currently, commonly used NMs include carbon-based materials, metals and metal oxides, silica, polymers, and nanozymes [15,16,17].

The nanotechnology holds significant potential for agricultural revitalization and food security [18,19]. The global agricultural nanoparticle market is substantial and is experiencing rapid growth. The largest and fastest-growing segments are currently nano-fertilizers and nano-pesticides, followed by plant growth regulators and biosensors, with the latter exhibiting the most explosive growth rate. NMs can be utilized in biosensors to detect contamination, diseases, or stress in plants, and also serve as delivery platforms for DNA or RNA, enabling genetic engineering in non-model plant species [20]. Organelle-selective targeted delivery using NMs as carriers has attracted widespread attention [21]. NMs enable precise delivery of fertilizers, pesticides, and genetic material, enhancing utilization efficiency and reducing environmental pollution [22,23]. Furthermore, NMs can improve the ability of plants to withstand stress by regulating essential physiological functions like photosynthesis and the activity of antioxidant enzymes, and by influencing microbial activities such as biofilm formation and hormone release [9,24]. NMs can activate stress signaling and defense pathways in crops, such as regulating the expression of related genes, increasing the content of resistance proteins, enhancing antioxidant enzyme activity, elevating chlorophyll levels, and promoting the synthesis of flavonoids [25,26]. Nanotechnology can also be integrated with other technologies such as the Internet of Things, big data analytics, artificial intelligence, and synthetic biology to precisely monitor crop growth status and material requirements. This enhances the accuracy of nutrient application and pest control, thereby improving crop growth and yield [27,28]. Simultaneously, it optimizes resource utilization and reduces the environmental impact of chemical inputs [29].

The detection of plant diseases, the precise delivery of fertilizers and pesticides, and the enhancement of stress resistance have greatly benefited agricultural practitioners [30,31]. This review critically examines the application of nanotechnology in advancing sustainable agriculture. Specifically, it (i) synthesizes current progress on nanomaterial-based biosensors for the detection of heavy metals, pesticide residues, antibiotics, and other environmental contaminants; (ii) evaluates the application of NMs as agrochemicals, including their role as fertilizers, pesticides, and growth stimulants; and (iii) assesses their potential in enhancing crop resilience against both biotic and abiotic stresses. By analyzing the advantages and limitations of these approaches, the review categorizes existing knowledge gaps and technological bottlenecks. The overarching objective is to provide a framework for integrating nano-enabled tools to address pressing agricultural challenges. Future research directions should focus on: (a) improving the safety, scalability, and field applicability of NMs; (b) integrating nanotechnology-driven systems for precision agriculture; (c) developing regulatory guidelines and risk assessment protocols to ensure environmental and food safety; and (d) advancing multifunctional platforms to mitigate the impacts of climate change, extreme weather events, and global food insecurity.

## 2. Scope and Methods

This review is based on a systematic literature search using the Web of Science Core Collection database, with a primary focus on research applications of nanotechnology in three areas: detection, agrochemicals, and stress resistance. The search timeframe was primarily limited to the last five years to capture the latest advancements in this rapidly evolving field. The retrieved records were subsequently screened to ensure their relevance to the review’s objectives. Finally, a comprehensive assessment of the full texts of potentially relevant articles was conducted to confirm their final inclusion in the review.

## 3. Nano-Enable Biosensors for Environmental and Agricultural Monitoring

With the development of the global economy and industry, an increasing number of chemicals are being applied in production and daily life. Consequently, environmental health and agricultural productivity are facing growing threats from complex pollutants. In agricultural environments, pollutants can include inorganic pollutants, organic pollutants, and emerging pollutants. Inorganic pollutants comprise heavy metals and metalloids, primarily originating from mining activities, industrial discharges, and the excessive use of chemicals. Organic pollutants are mainly synthetic agrochemicals, including insecticides, herbicides, pesticides, and fungicides. Emerging pollutants include antibiotics, microplastics, endocrine-disrupting compounds, and mycotoxins. These pollutants can disrupt the balance of ecosystems, and they can also be absorbed and accumulated by plants during growth, entering the human body through the food chain and causing irreversible harm to human health [32]. In addition to affecting the reproductive and immune systems, certain pesticides can also induce several metabolic diseases. Antibiotics are not only used for disease prevention and treatment but are also employed as veterinary growth promoters, which can lead to the development of antimicrobial resistance genes and even superbugs [33]. Agricultural toxins can cause water pollution, soil degradation, and loss of biodiversity, damaging natural ecosystems. Furthermore, human exposure may lead to cancer, kidney damage, endocrine disruption, reproductive effects, neurotoxicity, and developmental changes [34]. Bisphenols and chlorophenols are widely used as pesticides, disinfectants, and wood preservatives, posing neurotoxic, carcinogenic risks and causing damage to the endocrine and reproductive systems in humans [17].

Therefore, the development of effective methods for monitoring pollutants is essential in the field of environmental analysis. A biosensor is an analytical device that integrates a biological recognition element with a physicochemical transducer for quantitative or semi-quantitative analysis. Its core principle relies on the specific recognition of target analytes by biological molecules, which is then converted into a measurable signal, such as an electrical or optical signal [35,36]. The integration of NMs as recognition agents or signal transducers in biosensors offers advantages such as rapid response, high sensitivity, low cost, excellent selectivity, and real-time detection capabilities [33]. As a critical component of biosensors, NMs often possess high surface areas and exceptional physical, chemical, or electronic properties that enhance the extent of interaction and electron transfer rates [37]. Additionally, NMs can mimic natural enzymes by utilizing enzyme-like kinetics to convert substrates into products under physiologically relevant conditions. Compared to natural enzymes, these nanozymes are more stable, have broader applicability, and are more cost-effective [38]. Currently, the most extensively studied NMs include graphene, carbon nanotubes (CNTs), biochar (BC), quantum dots (QDs), carbon black (CB), metal nanoparticles, metal–organic frameworks (MOFs), and various composite materials [32,39].

**Figure 1:**
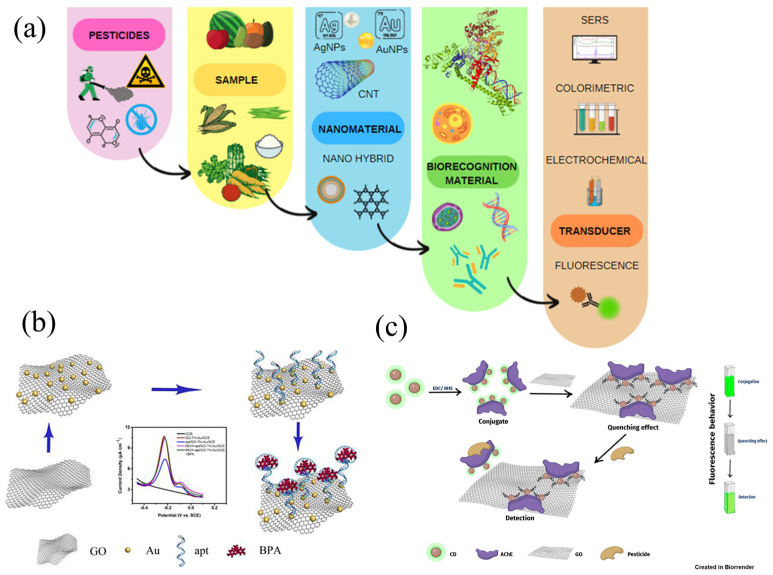
Schematic diagram of biosensor detection: (**a**) the necessary steps to construct biosensors, reprinted from ref. [50], (**b**) the specific recognition of bisphenol A on the surface of GO-Thi-Au nanoparticles modified electrode, reprinted from ref. [51], (**c**) fluorescent biosensor based on acetylcholinesterase and carbon dots-graphene oxide quenching test, reprinted from ref. [52].

In recent years, sensors based on various principles have been utilized for detecting a wide range of pollutants. These include electrochemical, colorimetric, fluorescent, photoelectrochemical (PEC), electrochemiluminescence (ECL), surface plasmon resonance (SPR), and surface-enhanced Raman spectroscopy (SERS) sensors [27,40,41]. They offer advantages such as rapid response, high sensitivity, low detection limits, minimal sample volume requirements, and often eliminate the need for sample pretreatment [42]. The application of NMs in electrochemical detectors (ECDs) can address limitations related to high overpotential, limited sensitivity, and restricted applicability. Additionally, a study has also reviewed the applications of wearable and implantable electrochemical sensors in plant health monitoring [43]. NMs act as catalysts for electrochemical reactions, enhance electron transfer, and improve conductivity [44]. Photoactive materials convert chemical information into detectable PEC signals through photoelectric conversion, with NMs offering high conversion efficiency and good biocompatibility [45]. SPR sensors can monitor molecular interactions between pollutants and plasmonic materials, where nanoparticles serve as effective sensing materials [34]. SERS is characterized by its simplicity, fast detection speed, high sensitivity, and excellent selectivity [46]. A schematic diagram illustrating various types of biosensors is presented in Figure 1.

Furthermore, the integration of nanosensors with artificial intelligence (AI) enables more accurate and effective interpretation of complex, multi-dimensional field data [47]. When combined with multispectral imaging visualization systems and algorithm libraries, this integrated approach allows for the automatic identification of plant stress throughout the entire growth cycle [48]. It provides a powerful tool for intelligent delivery systems, soil health promotion, and disease management [49]. The combination of biosensors with technologies such as AI and the Internet of Things facilitates real-time regulation of cultivation systems, thereby advancing precision agriculture aimed at minimizing resource requirements and maximizing crop yields.

### 3.1. Sensing and Quantification of Heavy Metals

The U.S. Environmental Protection Agency (EPA) classifies arsenic, mercury, lead, cadmium, chromium, and others as highly toxic heavy metals, which may cause organ damage, neurological disorders, and even cancer. Characteristics such as water solubility, non-biodegradability, and long-term bioaccumulation enable these metals to persist and accumulate in the environment and living organisms, amplifying their toxic effects through the food chain [53]. To safeguard environmental and human health, there is an urgent need to develop highly sensitive, rapid, low-cost, and portable heavy metal detection technologies.

Cellulose-MOFs (CelloMOFs) are functional materials formed by hybridizing biopolymers such as cellulose with metal–organic frameworks (MOFs), and they have been developed due to their advantageous properties [54]. Abdelhamid et al. successfully fabricated a hybrid material based on cellulose and hierarchical porous zeolitic imidazolate framework-8 (ZIF-8), termed CelloZIFPaper, using an aqueous-based process. This material combines high adsorption capacity with electrochemical sensing capabilities. When employed as a freestanding working electrode, CelloZIFPaper achieved a detection limit of 8 μM for Pb^2+^, demonstrating high selectivity and sensitivity [55].

Certain heavy metals can significantly inhibit the peroxidase-like activity of Co_3_O_4_ nanodisks and block electron transfer, resulting in a color change in the solution from blue to colorless. Leveraging this phenomenon, Zou et al. developed a highly sensitive colorimetric sensor for the rapid detection of Cd (II), Hg (II), Pb (II), and as residues in environmental water samples. This method enables multi-target detection with rapid operation, simplicity, high stability, and reproducibility; however, it cannot distinguish between the four specific types of heavy metals [56]. Liu et al. introduced a visual multiplex quantitative method based on a triple-channel V-Chip (TV-Chip) and DNA-nanoparticle probes. Using microfluidic technology to fabricate the TV-Chip and platinum nanoparticles (PtNPs) to catalyze the decomposition of H_2_O_2_ to produce oxygen, which drives the movement of an ink bar, the system achieved simultaneous visual quantitative detection of Cu^2+^, Pb^2+^, and Hg^2+^ for the first time [57].

Ramírez et al. developed an electrochemical affinity biosensor for the quantification of Pb (II) by integrating a glassy carbon electrode (GCE) with single-walled carbon nanotubes (SWCNTs) covalently modified with cysteine residues (Cys). They proposed a synergistic mechanism of “affinity enrichment–electrochemical detection,” which enables selective detection through Cys-Pb(II) affinity binding without the need for high cathodic potentials. The biosensor demonstrated high specificity and high recovery rates both in the presence of interferents and in real water samples [58]. Another study introduced a dual-signal surface-enhanced Raman spectroscopy (SERS) ratiometric strategy for detecting mercury ions (Hg^2+^) using Au@Ag/COF nanocomposites and Y-shaped DNA structures. The Au@Ag/COF nanocomposite provides strong electromagnetic field enhancement and high stability, while the Y-shaped DNA shortens the distance between Raman reporter molecules and the substrate surface, significantly improving detection sensitivity [46]. Furthermore, dual-signal SERS improves detection accuracy by simultaneously monitoring both “signal-off” and “signal-on” responses.

DNAzymes, as biomolecules, have emerged as promising recognition elements in sensors due to their high metal-binding affinity and specificity. The “8-17” DNAzyme undergoes self-cleavage of its substrate strand in the presence of Pb^2+^, leading to the disruption of conductive bridges between nanoparticles and a measurable change in electrical resistance. Leveraging this mechanism, a study developed a DNAzyme-functionalized biosensor based on platinum nanoparticles (PtNPs) for highly sensitive detection of Pb^2+^ [59]. Furthermore, Liang et al. integrated a DNAzyme-driven bipedal DNA walker with catalytic hairpin assembly (CHA) on an electrochemical platform. They utilized gold nanoparticle@zirconium-based metal–organic framework composites (AuNPs@Zr-MOF) loaded with methylene blue (MB) as a signal probe, achieving accurate and sensitive detection of Pb^2+^ [60].

### 3.2. Advanced Detection of Pesticide Residues

Agrochemicals have played a crucial role in enhancing the yield of food and agricultural products, meeting the basic food demands of the population [61]. However, residues of agrochemicals pose serious threats to both the environment and human health, potentially leading to cancer, neurological damage, reproductive and developmental disorders, among other issues [34]. Currently, pesticide residues have been detected in food products, with some instances exceeding the maximum residue limits by as much as 20% [62]. Therefore, it is imperative to develop rapid and effective methods for pesticide detection.

Electrochemical biosensors are low-cost and portable. Arduini et al. developed a paper-based multifunctional sensor that enables the quantitative detection of multiple pesticides (paraoxon, 2,4-dichlorophenoxyacetic acid, and atrazine) by monitoring changes in electrochemical signals resulting from the inhibition of specific enzymes, such as butyrylcholinesterase, alkaline phosphatase, and tyrosinase [63].

Electrochemiluminescence, which combines electro-oxidation/reduction and chemiluminescence processes, is widely used in immunoassays, detection probes, and imaging target transduction. NMs can function as co-reactant catalysts or luminophore carriers in ECL systems. Upon electrical excitation, they are promoted to an excited state and subsequently emit light upon returning to the ground state. Li et al. modified multi-walled carbon nanotubes (MWCNTs) with 2,2,6,6-tetramethylpiperidine-1-oxyl (TEMPO) to fabricate a TEMPO-MWCNTs nanomaterial-modified electrode (TEMPO-MWCNTs/GCE). This electrode exhibited excellent electrocatalytic activity, enabling efficient catalysis of the oxidation/reduction reactions of H_2_O, luminol, H_2_O_2_, and O_2_ [64]. Furthermore, by immobilizing acetylcholinesterase (AChE) and choline oxidase (ChOx) on the TEMPO-MWCNTs/GCE, an ECL sensor was constructed for the highly efficient and sensitive detection of malathion (MA) [64]. The multifunctional electrocatalytic properties of TEMPO-MWCNTs provide a new direction for the further development of ECL technology, with potential future applications extending to the detection of other biomarkers or environmental contaminants.

In recent years, nanozymes have been widely employed in biosensing as a means of signal transduction and amplification. They can function either directly or in conjunction with biomolecules to develop potential pesticide sensing strategies [38]. To detect environmental levels of acetamiprid (ACE), Yu et al. developed an aptasensor based on Fe–N–C single-atom nanozymes (SAzymes). This sensor utilizes the specific binding between the aptamer and the target molecule to modulate the catalytic activity of the nanozyme, enabling highly efficient detection of acetamiprid through a simple and low-cost method [65].

### 3.3. Antibiotic Residue Monitoring in Agroecosystems

Antibiotics have been classified as emerging contaminants, garnering significant public and scientific concern. They are extensively used as therapeutic agents and growth promoters in animal husbandry. However, a substantial proportion of antibiotics are not absorbed by humans or animals and are excreted into the environment. This leads to the disruption of microbial communities and the proliferation of antibiotic resistance genes, posing a substantial threat to human health [37].

Using rice husk residue and lysine as the carbon and nitrogen sources, respectively, Qi et al. developed a nitrogen-doped carbon quantum dot (N-CQDs) fluorescent probe. The results demonstrated that the N-CQDs exhibit high selectivity and were successfully applied to detect Fe^3+^ and tetracycline antibiotics (TCs) in real water samples, including tap water and river water. Moreover, due to their low toxicity and good biocompatibility, the N-CQDs can be used for fluorescent imaging of Fe^3+^ and TCs in living cells [66]. The physisorption strategy is widely employed as a general sensing approach in fluorescent platforms. However, it suffers from limitations such as non-specific probe displacement, low desorption efficiency, and the poor affinity of emerging two-dimensional materials for DNA, which restrict its application in fluorescence sensing. To address these issues, Tan et al. constructed a two-dimensional MOF-DNA nanosensor using a short-chain FAM-labeled DNA as the signal reporter. By leveraging surface passivation and covalent linkage strategies, they achieved highly sensitive fluorescence detection of oxytetracycline (OTC) [67].

A systematic evaluation was conducted to assess the impact of two-dimensional (2D) materials on the performance of surface plasmon resonance (SPR) sensors. It was found that the thickness of the 2D material exhibited an optimal value. Beyond this value, increased energy loss occurred, resulting in diminished sensitivity. For MoS_2_, a thickness of 5 nm led to a 24% enhancement in sensitivity and achieved a detection limit as low as 0.05 µg L^−1^, which was 12 times lower than that of the bare gold chip system. The MoS_2_-enhanced SPR sensor demonstrated high sensitivity and a low detection limit in the detection of sulfonamide antibiotics [68].

Aptamer-based sensors are also widely used for antibiotic detection. Wang et al. proposed an electrochemical aptasensor for the highly selective and sensitive detection of florfenicol (FF). The study utilized a MOF-derived Mn, N co-doped Co–C nanomaterial (CoMnN–Cs) as the electrode modification material, in combination with gold nanoparticles (AuNPs) and exonuclease I (Exo I) to achieve dual signal amplification. Meanwhile, trimetallic nanoparticles (PtPdCuNPs) loaded with thionine (Thi) were employed as a signal probe to further enhance the electrochemical response [69]. In another study, Sun et al. constructed a photoelectrochemical aptasensor by forming helical TiO_2_@MoS_2_ nanoarrays on FTO conductive glass, followed by the chemical immobilization of a thiolated aptamer (Aptamer-SH). The covalent bonding between the S atoms of MoS_2_ and the thiolated aptamer allowed specific recognition of chloramphenicol (CAP). The binding of CAP resulted in a reduction in photogenerated carriers and a decrease in photocurrent, enabling rapid and highly selective detection of CAP [70]. Additionally, there have been reports on the use of photoelectrochemical biosensors for CAP detection. For instance, Liu et al. synthesized Se-doped In_2_S_3_ (In_2_S_3−x_Se_x_) NMs, in which selenium doping introduced defect states that significantly enhanced the light absorption efficiency and the separation efficiency of photogenerated carriers [71].

### 3.4. Biosensing of Emerging Contaminants and Complex Pollutants

In addition to the pollutants mentioned above, a wide range of toxic and hazardous substances that are resistant to degradation and pose significant threats to human health persist in the environment. These include persistent organic pollutants (POPs), hazardous chemicals, endocrine-disrupting compounds, and agricultural toxins. Therefore, it is imperative to develop efficient, accurate, and cost-effective detection methods for these contaminants.

Polychlorinated biphenyls (PCBs), as a class of persistent organic pollutants (POPs), pose significant threats to the environment and human health. Cui et al. synthesized functionalized silver nanoparticles (Ag NPs) using β-cyclodextrin (β-CD), which served simultaneously as a reducing agent and a surface modifier. Based on this material, they developed a dual-mode sensor utilizing both surface plasmon resonance light scattering (SP-RLS) and surface-enhanced Raman scattering (SERS), enabling highly sensitive detection of PCBs [72].

Substances such as hydroquinone (HQ) and catechol (CC) are widely used in various industrial fields. However, they are harmful environmental pollutants due to their slow degradation and high toxicity. To enable efficient and sensitive detection of HQ and CC in environmental water, a molybdenum oxide nanocomposite (MoO_3_@KSC) was synthesized using chicken feather waste as a carbon source and applied for electrochemical detection. This sensor exhibited excellent selectivity, reproducibility, and stability [73]. An electrochemical tyrosinase biosensor based on amino acid ionic liquid (AAIL)-functionalized graphene was developed for the detection of catechol. It achieved a detection limit as low as 8 nM, with a linear range from 25 nM to 11,100 nM. When applied to real water samples, the recovery rate ranged from 90.5% to 108.6%, demonstrating high sensitivity, repeatability, inter-electrode reproducibility, selectivity, and long-term stability. The AAIL effectively improved the dispersion of hydrophobic graphene in aqueous media while preserving the excellent electrical conductivity and structural integrity of graphene [74].

Endocrine-disrupting compounds (EDCs) pose potential hazards to both the environment and human health. Bisphenol A (BPA), a common EDC, is widely present in plastic products. Using tyrosinase (Tyr) as a model enzyme, Wu et al. immobilized it on a graphdiyne (GDY)-modified glassy carbon electrode (GCE) to construct a Tyr-GDY-Chi/GCE biosensor [75]. Similarly, Ma et al. encapsulated Tyr within a copper–porphyrin MOF nanofilm to develop a Tyr@Cu-TCPP biosensor. Both sensors demonstrated high recovery rates, excellent selectivity, and good stability in the detection of BPA in real samples [76]. In a separate approach, Povedano et al. designed an electrochemical enzymatic biosensor based on reduced graphene oxide–rhodium nanoparticles (rGO–RhNPs) for the detection of trace 17β-estradiol in environmental and clinical samples. The Rh nanoparticles were generated in situ and deposited on the rGO surface, followed by the immobilization of laccase via glutaraldehyde cross-linking [77].

Mycotoxins are low-molecular-weight secondary metabolites produced by fungi, known for their hepatotoxicity, teratogenicity, carcinogenicity, and mutagenic effects, which pose serious risks to human health. Conventional detection methods are often time-consuming, costly, require complex pretreatment procedures and professional operation, making them unsuitable for on-site rapid detection [78]. Among these toxins, zearalenone (ZEN), a mycotoxin produced by Fusarium species, widely contaminates cereals and liquid food products. Moradi et al. developed a nanofiber-modified graphite electrode using electrospinning technology for the rapid and highly sensitive detection of ZEN in food simulants [79].

### 3.5. Summary and Discussion

In the construction of biosensors, the application of nanotechnology modifications can contribute to aspects such as recognition capability, signal transduction, detection limit, specificity, or broad-spectrum performance, thereby enhancing detection accuracy, sensitivity, stability, and biosafety. Biosensors employ diverse detection methods, each with its own advantages. Colorimetric detection is intuitive and convenient, allowing direct visual observation; methods such as SPR, SERS, and fluorescence offer high sensitivity and low detection limits but often require large, sophisticated instruments; electrochemical and photochemical detection methods are accurate and sensitive, holding promise for the development of portable sensors. The applications of various biosensors have been shown in Table 1. Future research on sensors may focus on the following aspects: (1) improving the ability of recognition units to distinguish target substances in complex systems and enabling simultaneous multi-target detection; (2) developing NMs with specificity, biocompatibility, and environmental friendliness; (3) designing portable sensors that require minimal sample preprocessing, eliminate the need for long-distance storage and transportation, and allow on-site testing; (4) integrating detection devices with AI, network, or communication technologies to quantify results into digital signals.

## 4. Nanomaterials as Smart Agrochemicals for Sustainable Crop Production

To support global agricultural production for a growing population, farmers have resorted to the excessive use of agrochemicals to reduce crop diseases and pests and enhance crop yield [88]. However, with conventional application methods, only a small fraction of the active ingredients reaches the target crops, approximately 30–55% for nitrogen-based fertilizers, 18–20% for phosphorus-based fertilizers, and 30–40% for pesticides [89]. The majority of agrochemicals are released into the environment, leading to soil degradation, groundwater contamination, water eutrophication, and other forms of ecological imbalance, while also causing significant economic losses. Therefore, it is imperative to develop novel approaches that improve the utilization efficiency of agrochemicals, ensuring a safe, effective, and sustainable means of meeting food supply demands.

**Figure 2:**
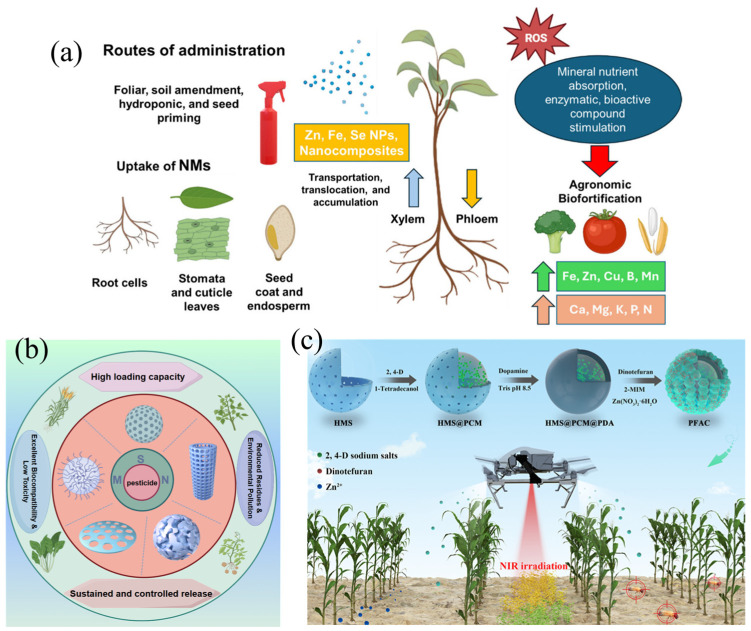
Applications of NMs as agrochemicals: (**a**) mechanism of NMs strengthening crops, reprinted from ref. [97], (**b**) key characteristics of NMs for pesticide delivery, reprinted from ref. [98], (**c**) controlled and targeted delivery of agrochemicals enabled by photothermal properties, environmental responsibility, and deep learning, reprinted from ref. [99].

Recently, nanotechnology has been extensively explored for applications in plant systems, such as nano-fertilizers, nano-pesticides, and nano-promoters [90]. NMs enable the slow or stimuli-responsive release of active ingredients in agrochemicals, providing more stable and targeted effects on crops and thereby enhancing productivity [91,92,93]. Commonly used types of NMs include mesoporous silica nanoparticles (MSNs), metal nanoparticles (e.g., silver nanoparticles), peptide-based NMs, carbon nanotubes (CNTs), and carbon dots, etc. [21,94,95,96]. Figure 2 illustrates research on the application of NMs as various types of agrochemicals. The application of nanomaterials in agrochemicals has been shown in Table 2.

### 4.1. Nano-Fertilizer: Precision Nutrients Delivery

Currently, synthetic fertilizers are produced using finite mined resources, making their manufacturing and application unsustainable. Moreover, their excessive use leads to environmental pollution and resource wastage. Nano-fertilizers, owing to their high efficiency and precision release properties, are regarded as a crucial solution for sustainable agriculture [1]. By enabling targeted delivery and controlled release of nutrients, nano-fertilizers can significantly enhance nutrient use efficiency while reducing pollution in soil and water bodies [100]. The application of NMs as soil amendments can significantly improve soil properties. They enhance water and nutrient retention capacity, increase soil organic carbon content, and strengthen soil cohesion [101].

Conventional urea fertilizers release nutrients too rapidly, with only about 50% of the nitrogen being effectively utilized by crops. The remainder is lost through volatilization, soil reactions, and leaching, leading to significant resource waste and environmental pollution. To address this issue, a nanocomposite composed of GO loaded with urea (termed N-doped graphene oxide, urea@GO) has been developed to slow the release rate and improve nitrogen use efficiency [102]. In another study, Zhang et al. designed a sustained-release fertilizer structured with a nano-carbon (CN) core, aminated mesoporous silica (mSiO_2_-NH_2_) as the shell for urea loading, and a polydopamine (PDA) coating, denoted as CN@mSiO_2_-NH_2_@Urea@PDA. When applied as a substitute for 40% of the conventional base fertilizer, this material increased flowering Chinese cabbage yield by 69.94% [103].

Nitrogen-doped carbon dots (N-CDs) were prepared from citric acid and 1,3-diaminopropane, which promoted the growth and nutritional quality of lettuce. By increasing electron transport rate (ETR), photochemical quenching, light energy conversion efficiency of photosystem II and Rubisco enzyme activity, N-CDs promoted the efficiency of photosynthesis and the accumulation of photosynthetic products [104].

Zinc (Zn) is an essential trace element for both plant and human health. Application of Zn fertilizers is an effective strategy to enhance Zn concentrations in crops. Zn-doped Mg-Fe layered double hydroxides (Zn-doped Mg-Fe LDHs) was designed as a new type of zinc fertilizer. The release of Zn is controlled by pH, which helps to solve the problem of soil Zn deficiency and improve crop yield and nutritional quality [105].

Research has demonstrated that nano-CuO can reduce the excessive release of copper and, at low doses, shows no negative effects on plant growth and reproduction. It may also undergo biotransformation, thereby reducing its toxicity [106]. However, He et al. reported that wheat exposed to CuO-NPs and Ce_2_-NPs for 21 days showed contrasting effects. CuO-NPs caused oxidative stress, disrupted nitrogen metabolism, and reduced growth by ~15%, with plant accumulation nearly 20-fold higher than Ce_2_-NPs due to greater solubility [107].

Bio-based polymer-coated controlled-release fertilizers are eco-friendly and cost-effective but often suffer from poor nutrient release control due to hydrophilicity and microporosity. To overcome this, Yang et al. developed a dual-modified all-biobased polyurethane-coated urea (DHBCU) with siloxane and copper laurate (LAC), achieving superhydrophobicity, enhanced elasticity, and a prolonged nitrogen release period to 112 days while reducing NMs inputs [108]. Similarly, Zhang et al., synthesized nano-sized copper laurate (NLAC), exhibiting superhydrophobic, superoleophilic, and antimicrobial properties, with great potential for applications in controlled-release fertilizers and oil-water separation, alongside economic and environmental benefits [109].

### 4.2. Nano-Pesticide; Targeted Pest and Pathogen Suppression

Agrochemicals have been extensively used to enhance productivity; however, their excessive application poses significant threats to environmental and human health [7]. Due to their unique size effects, NMs demonstrate superior comprehensive efficacy in controlling crop diseases compared to ionic forms, bulk particles, and commercial pesticides, enabling direct suppression of pest and pathogen growth [110]. Consequently, a wide range of NMs have been developed to improve the photochemical stability, bioactivity, controlled-release properties, and environmental compatibility of pesticides [111,112,113,114]. The comparison of various nano pesticide delivery systems is shown in Table 3.

Santana et al. designed a multifunctional quantum dot (QD) modified with β-cyclodextrin (β-CD) on its surface. The QD was functionalized using the biomolecular recognition motif RbcS guiding peptide, enabling specific targeting via the translocon on the outer chloroplast membrane. This innovation provides a non-transgenic precision tool for plant biology research and can also be utilized for efficient delivery of pesticides or nutrients [115].

The insecticide Emamectin Benzoate (EB) was encapsulated within β-CD and amino-functionalized zein (AM-zein) to form a core-double shell structure EB@β-CD/AM-zein, which improved the encapsulation and photostability of EB, and enhanced the leaf affinity and dispersibility [116].

To address the issue of initial burst release associated with conventional avermectin (AV), a pH-responsive controlled-release formulation was developed using AV as a model pesticide and MXene (Ti_3_C_2_) as the carrier. The resulting AV@Ti_3_C_2_ complex demonstrated sustained insecticidal activity and enhanced biosafety [117]. In another approach, avermectin (AVM) was encapsulated within composite carrier to form AVM@P-zein/CMC-g-PDMDAAC nanoparticles. This system achieved a high encapsulation efficiency of up to 82.11%, and offered multiple advantages, while maintaining the pesticide’s bioactivity [118].

The biocompatibility of nanocarrier materials is also of critical importance. Liu et al. focused on utilizing starch nanocrystals (SNCs) as nanocarriers to develop an intelligent pesticide formulation based on thiamethoxam (TMX), designated SNC@TMX. This system successfully enhanced the stability and sustained-release properties of TMX while reducing its toxicity to bees [119]. In a separate study, biocompatible sodium alginate (SA) was employed as a hydrophilic carrier to form core–shell structured NPs, which significantly enhanced the photostability and insecticidal efficiency of Phloxine B (PB) and enabled its controlled release within target pests [120].

Novel nano-pesticides characterized by high efficiency, low toxicity, and environmental friendliness are not only applied for pest control but also for managing phytopathogens. Han et al. constructed a nanoplatform using a Cu-TCPP MOFs as the carrier to load diniconazole (DIN), resulting in Cu-TCPP@DIN@MPN nanoparticles. This system significantly enhanced pesticide utilization and environmental adaptability through multidimensional regulation, while ensuring biosafety [121]. Huang et al. developed a pH-responsive multifunctional nano-pesticide-fertilizer complex (Pro/DA-6@ZIF-8@siRNA), integrated the chemical pesticide propiconazole (Pro), a nucleic acid pesticide and the plant growth regulator diethyl aminoethyl hexanoate (DA-6) to synergistically control rice sheath blight, while simultaneously promoting rice growth [122]. In another study, Liu et al. developed a nano-pesticide designated as [dsRNA-Luvangetin]@CQAS, which can be tailored to target different pathogens by adjusting the dsRNA sequence. This system combines antibacterial activity, gene silencing, and immune activation functions, making it suitable for integrated disease management [123].

Conventional pesticides with single-site modes of action are highly prone to inducing drug resistance. NMs can inhibit the development of pathogen resistance through multi-modal mechanisms. Research has demonstrated that the combined use of Cu-NPs with conventional fungicides can reduce fungicide dosage and suppress resistance to *B. cinerea* [124].

### 4.3. Nano-Bio-Stimulants and Growth Promoters

Plant biostimulants do not function as nutritional sources but rather as physiological regulators that enhance plant growth and development through biostimulation and seed priming. Concurrently, biofortification has been employed as a complementary strategy to produce “healthier food” by cultivating nutritionally enhanced crops through modern biotechnology, conventional plant breeding, and improved agronomic practices [125]. At non-toxic concentrations, NMs can deliver essential elements to support plant growth and metabolism, thereby enhancing developmental vigor and antioxidant capacity. They can also facilitate the transport of appropriate amounts of minerals such as zinc, iron, manganese, potassium, and selenium to edible tissues, increasing the content of micronutrients, macronutrients, and bioactive compounds in crops [97].

Selenium (Se) not only promotes crop growth but also helps fulfill human dietary selenium requirements by enhancing its concentration in food products [126]. *Caralluma tuberculata* exhibits low seed germination rates and slow rooting under natural conditions. The application of selenium nanoparticles (SeNPs) enhanced plant stress resistance by modulating the antioxidant system and secondary metabolic pathways. A low concentration of SeNPs (100 µg L^−1^), combined with indole-3-butyric acid (IBA), significantly promoted in vitro rooting and growth of *C. tuberculata* [127]. The SeNPs were rapidly converted into Se (IV) and organic selenium within the plant and specifically bound to two selenium-binding proteins. Furthermore, SeNPs up-regulated genes associated with plant defense, growth, and antioxidation. They also alleviated oxidative stress promoting plant growth and enhancing overall plant health [128].

Seeds of rice (*Oryza sativa* L.) were treated with green-synthesized nano-scale zerovalent iron (G-nZVI), produced using pomegranate peel extract, as a nanopriming agent. This treatment significantly enhanced seed germination, improved seedling vigor, and increased grain yield [129]. Lahiani et al. synthesized single-walled carbon nanohorns (SWCNHs) and applied them to plants, including both crops and tobacco cell cultures. The treatment significantly accelerated seed germination in multiple crop species, increased stem length and biomass, and promoted the growth of tobacco cells [130].

The application dosage of nano-enhancers is a critical factor requiring careful consideration. When GO was applied as a growth promoter to mung beans, a concentration of 1200 mg L^−1^ significantly increased the activities of superoxide dismutase (SOD), peroxidase (POD), and catalase (CAT), alongside elevated chlorophyll content and biomass. However, at a higher concentration (1500 mg L^−1^), the antioxidant system was likely overwhelmed, leading to oxidative damage and inhibited growth [131]. Furthermore, other studies have demonstrated that GO at high concentrations significantly suppresses shoot biomass and shoot elongation in rice seedlings. GO induced acidification of the nutrient solution, triggering oxidative stress and generating excessive reactive oxygen species (ROS), which subsequently caused lipid peroxidation and ultimately inhibiting plant growth [132]. In addition to promoting plant growth at low concentrations, GO can also safely and effectively enhance fruit quality. When a GO solution was injected into the stems of watermelon plants, the average fruit circumference increased by 5 cm, and the sugar content rose from 10.16 Brix to 11.73 Brix [133].

### 4.4. Integration of NMs in Smart and Sustainable Crop Management

The development of nano-fertilizers and nano-pesticides has brought new impetus to agricultural production. However, challenges persist, including single functionality in controlled release systems, limited regulation methods, issues of non-biodegradable toxicity, and insufficient targeting precision. To optimize the use of agricultural chemicals, improve sustainability, reduce energy waste and negative environmental impacts, and meet the requirements of precision agriculture, it is essential to integrate novel technologies such as sensors, GPS, robotics, drones, and artificial intelligence with innovations in agricultural chemistry. This integration will facilitate highly efficient and sustainable agricultural management.

Ji et al. developed a pesticide-fertilizer all-in-one nanoplatform (PFAC) using HMS@PCM@PDA@ZIF-8 for intelligent agrochemical delivery. NIR-triggered heating released the herbicide, while acidic conditions decomposed ZIF-8 to release the insecticide and zinc. Coupled with YOLO-v3 drone imaging, this system enabled automated weed detection and precision crop management [99].

### 4.5. Summary and Discussion

The integration of nanotechnology with agricultural agents such as fertilizers and pesticides can effectively enhance the utilization efficiency of active ingredients while reducing environmental impact [134,135]. Based on their mechanisms of action, they are categorized into slow-release, controlled-release, and bioactivation. Slow-release functions by delaying the release rate of agricultural agents; controlled-release precisely matches crop needs by responding to conditions such as pH, temperature, or humidity; and bioactivation utilizes microorganisms to decompose insoluble nutrients [136]. Plant growth promoters play a role in stimulating seed germination, enhancing crop growth vitality, or improving crop nutritional content. However, current research still has limitations. Future studies could expand field trials to investigate the practical effects of large-scale applications. Secondly, exploring the mechanisms of nanomaterial interactions within plants could facilitate precise nutrient delivery. Additionally, attention should be paid to the impact of combined exposure to multiple NMs on crop growth and health.

**Table 2:** Application of nanomaterials in fertilizers, pesticides, and enhancers (↑ is increase and ↓ is decrease).

	Nanomaterials	Crop/Target	Dosage	Effect	Mechanism	Reference
Nanofertilizers						
1	CeO_2_-NPs	Wheat (*Triticum aestivum*)	100 mg L^−1^	Carbohydrate-related metabolic pathways ↑, Antioxidant capacity ↑, Energy metabolism ↑	Starch and sucrose metabolic pathways ↑, Energy storage ↑, Plant growth ↑	[107]
2	Zn-doped Mg-Fe-LDHs	Barley (*Hordeum vulgare* cv. Antonia)	10 mg pot^−1^	Zn concentrations ↑	Organic acids ↑, phytosiderophores ↑	[105]
3	ternary nanocomposite UF/PBS/potassium dihydrogen phosphate (MKP)	-	-	Slow-release performance ↑, Controlled-release properties ↑	hydrogen bonding interactions and a “cage effect”	[137]
4	N-CDs	Lettuce (*Lactuca sativa*)	100 and 200 mg L^−1^	Biomass accumulation ↑, nutrient content ↑	Actual photosynthesis rate ↑, Activity of glutamine synthetase ↑	[104]
5	Urea@GO	-	150 mg L^−1^	Slow releas	pH, concentration of urea, presence of water, solar radiation, graphene sheets, etc.	[102]
6	CN@mSiO_2_-NH_2_@Urea@PDA	*Brassica rapa*	-	Slow release, Choy sum yield ↑, Nitrogen utilization Efficiency ↑, Nitrogen loss ↓	Material’s porosity decreases ↓	[103]
7	DHBCU	-	-	Hydrophobicity ↑, Elasticity ↑, Controlled-release performance ↑, Nitrogen release period ↑	Water penetration ↓, Nutrient release ↓	[108]
Nanopesticides						
1	AV@Ti_3_C_2_	*Maize*	1.5 g soil mixed with 0.25 g AV@Ti_3_C_2_	Slow release	pH-responsive slow-release	[117]
2	AVM@P-Zein/CMC-g-PDMDAAC	Cucumber (*Cucumis sativus*)	35.95 mg L^−1^ (LC_50_)	Stability ↑, Drug loading ↑, Anti-ultraviolet ↑, Adhesion ↑, Sustained release ↑, Toxicity ↓	Phosphorylation + Electrostatic coating, UV shielding/adhesion	[118]
3	EB@β-CD/AM-Zein	Diamondback moth (*Plutella xylostella* L.)	1.78 mg L^−1^ (LC_50_)	Encapsulation efficiency ↑, Insecticidal activity remained stable	α-amylase in insect guts can degrade the inner β-CD shell	[116]
4	SNC@TMX	*Diaphorina citri* (Hemiptera: Liviidae)	1.562–50 mg L^−1^	Insecticidal activity ↑	Pesticide utilization efficiency ↑, Toxicity to non-target organisms ↓	[119]
5	Esterase/GSH	Sf9 insect cells	-	Cytophototoxicity ↑	pest-specific esterase-6 ↑, Intracellular GSH levels ↑	[120]
6	Pro/DA-6 @ZIF-8 @siRNA nanoparticles (Nps)	*Rhizoctonia solani*	0.125 mg L^−1^	Efficacy of non-invasive pesticides ↑, Rice seedling growth ↑	Fungal-secreted acidic substances trigger the breakage of Zn^2+^-N coordination bonds in ZIF-8	[122]
7	[dsRNA-Luvangetin]@CQAS	*Sclerotinia sclerotiorum*	200 mg L^−1^	*Sclerotinia sclerotiorum* ↓, Co-infection of both viruses and fungi ↓	Chemical fungicidal activity, gene silencing, and plant immune activation	[123]
8	Cu-TCPP@DIN@MPN	*Fusarium oxysporum*	10 mg mL^−1^	Inhibition of *Fusarium oxysporum* ↑, photostability ↑	Antibacterial activity ↑	[121]
Bio-Enhancers (Growth Promoters and Seed Priming Agents)						
1	SeNPs	*Arabidopsis thaliana*	5–100 μg plant^−1^	Selenium content ↑	SeNPs were biotransformed into selenium (IV) and SeMet	[128]
2	SeNPs	*Caralluma tuberculata*	0.100 mg L^−1^	Rooting frequency ↑, Number of roots ↑, Fresh weight ↑, Dry weight ↑	Antioxidant enzyme activity ↑	[127]
3	G-nZVI	rice (*Oryza sativa* L. cv. Gobindobhog)	80 mg L^−1^	Seed germination ↑, Seedling vigor ↑, Hydrolytic enzyme activity ↑, Plant height ↑, Tiller count ↑, Panicle weight ↑, Overall yield ↑	Activity of antioxidant enzymes ↑, Photosynthetic efficiency ↑	[129]
4	GO	Mungbean (*Vigna radiata* L.)	1200 mg L^−1^	Length of roots and shoots ↑, Number of leaves, root nodules per plant, pods and seeds per pod ↑	Activating the antioxidant system	[131]
5	GO	Watermelon	10 mg L^−1^	Perimeter ↑, sugar content ↑	Expression of auxin- and cytokinin-related genes	[133]
6	SWCNHs	barley, corn, rice, soybean, tomato, switchgrass	25, 50, 100 mg L^−1^	Seed germination ↑, growth of tobacco cells ↑	Altered the expression of genes related to stress response, cell growth, and metabolic processes in crops	[130]

**Table 3:** Comparative analysis of nano-agrochemical delivery systems.

Category	Nanocarrier Type	Active Ingredient	Controlled Release Behavior and Triggers	Dose Reduction/Efficacy Increase	Phytotoxicity	References
Nutrient Delivery	Mesoporous Silica (CN@mSiO_2_-NH_2_)	Urea	Slow Release: 24% decrease in release rate Trigger: Diffusion control (PDA coating reduces porosity)	21.64% higher N utilization efficiency, 69.94% yield increase	Significant growth promotion in *Brassica rapa*	[103]
Nutrient Delivery	Polymer/Hydrogel (UF/PBS/MKP)	N, P, K	Slow Release: Excellent N slow-release performance Trigger: Diffusion via hydrogen bonding and “cage effect”	Indirect reduction via improved utilization efficiency	-	[137]
Nutrient Delivery	Polymer (DHBCU)	Urea	Highly Slow Release: Release period extended to 112 days Trigger: Superhydrophobic layer inhibits water penetration	Greatly extended efficacy, reduces top-dressing frequency	-	[108]
Pesticide Delivery	MOF (ZIF-8)	Propyrisulfuron (Pro), DA-6, siRNA	Smart/Targeted Release: Fungal-secreted acids trigger carrier degradation Trigger: pH	Enhanced efficacy of non-invasive pesticides, promoted rice seedling growth	Promoted rice seedling growth, no phytotoxicity	[122]
Pesticide Delivery	Polymer (Zein-based)	Abamectin (AVM)	Slow/Smart Release: Sustained release, anti-UV Trigger: pH-triggered release	High drug loading, LC_50_ of 35.95 mg L^−1^	Effective against target pest (diamondback moth)	[118]
Pesticide Delivery	Polymer (β-CD/AM-Zein)	Emamectin Benzoate (EB)	Smart/Targeted Release: α-amylase in insect guts degrades β-CD shell Trigger: Enzyme-triggered	High encapsulation efficiency, LC_50_ of 1.78 mg L^−1^, stable insecticidal activity	Effective against target pest (diamondback moth)	[116]
Biopesticide Delivery	Organic Polymer/Lipid ([dsRNA-Luvangetin]@CQAS)	dsRNA, Luvangetin	Synergistic Effect: Triple synergy of chemical fungicide, gene silencing and plant immunity activation Trigger: Not specified, presumed release upon contact/uptake	Effective against Sclerotinia sclerotiorum, suppresses viral and fungal co-infection	Safe on solanaceous crops	[123]
Pesticide Delivery	Nanoclay/LDH (AV@Ti_3_C_2_)	Abamectin (AV)	Highly Slow/Smart Release: Only 12.35% cumulative release in soil after 14 days Trigger: pH-responsive	Maintained 65% mortality rate while minimizing burst release and loss	Safe on maize	[117]

## 5. Nano-Enabled Approaches for Enhancing Crop Stress Tolerance

**Figure 3:**
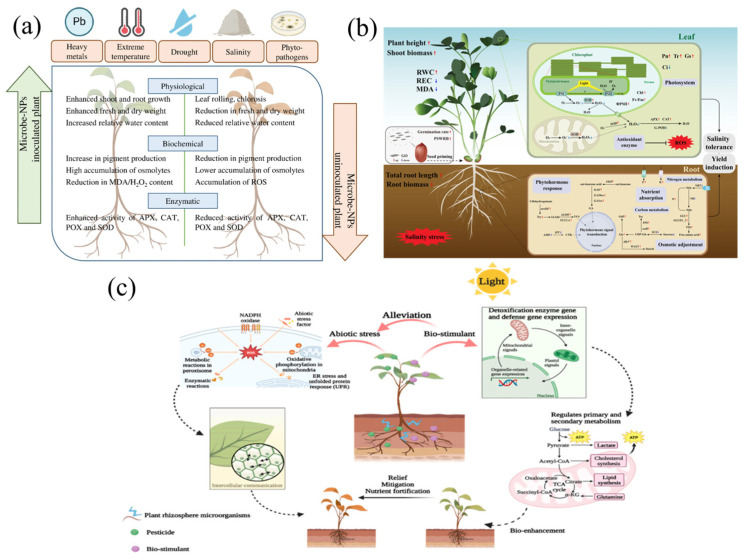
Applications of nanomaterials in crop stress resistance: (**a**) the synergistic effect of microorganisms-nanoparticles on plants under stress, reprinted from ref. [135], (**b**) GO promotes peanut seed germination and enhances salt tolerance in seedlings, reprinted from ref. [142], (**c**) effects of abiotic stress on crop health, reprinted from ref. [143].

Currently, challenges such as climate change, heavy metal pollution, plant pathogens, crop infectious diseases, and reduced crop yields threaten agricultural sustainability [88]. Both biotic and abiotic stresses reduce crop productivity by impairing plant growth and development in the field and by degrading crop quality during storage [20]. When exposed to stress conditions, plants tend to synthesize and accumulate defense-related phytohormones to ensure survival under adverse conditions [138]. Plants recognize pathogens or physical damage through signaling pathways involving Pattern-Triggered Immunity (PTI) and Effector-Triggered Immunity (ETI) systems. PTI serves as the first line of plant defense, which includes calcium ion (Ca^2+^) influx, reactive oxygen species (ROS) production, and the regulation of hormones such as ethylene (ET), abscisic acid (ABA), salicylic acid (SA), and jasmonic acid (JA). If PTI fails, ETI is activated [139]. Notably, NMs can induce or enhance similar protective mechanisms in plants, thereby strengthening stress resistance. Meanwhile, NMs can promote plant growth, alleviate oxidative stress, induce defense responses, and modulate plant-microbe interactions [140]. Due to their small size, NMs can readily enter cells and influence physiological and biochemical processes [135]. Nanotechnology has emerged as a feasible strategy to enhance plant resilience under unfavorable conditions and improve overall performance [141]. Figure 3 illustrates the application of different NMs in crop stress resistance.

### 5.1. Mitigation of Abiotic Stress

Abiotic stress refers to the adverse effects on crops caused by non-living factors such as temperature, salinity, drought, and heavy metals. The extent of its impact depends on the type and intensity of the stress, plant species, stress combinations, and the developmental stage of the plant [141]. Abiotic stress is one of the critical constraints affecting agricultural production and can lead to substantial reductions in both crop yield and quality [144]. NMs can help plants adapt to unfavorable conditions by modulating reactive oxygen species, stress-related gene expression, and signaling pathways [145].

Under drought stress, the cellular redox homeostasis in crops is disrupted, leading to excessive accumulation of reactive oxygen species (ROS), which damages cellular structures and further impairs key metabolic pathways such as photosynthesis and water metabolism [146]. Zhao et al. treated drought-stressed soybean (*Glycine max* L.) with GO, which significantly increased the relative water content (RWC) in stems, leaves, roots, and soil. GO promoted root development, enhanced the antioxidant defense system, and alleviated oxidative damage. By upregulating the expression of drought-responsive genes (GmP5CS, GmGOLS, GmDREB1, GmNCED1) and increasing the levels of ABA, SA, JA, and auxin (IAA), GO improved drought tolerance in soybean [147].

Under drought conditions, maize treated with Zeolitic Imidazolate Framework-8 (ZIF-8) NMs exhibited a 57.9% increase in root length, an 11.9% increase in stem length, and a 44.8% increase in root tip number. Mechanistic studies revealed that ZIF-8 treatment activated glycerophospholipid metabolism and sugar metabolism pathways. Additionally, ZIF-8 captured electrons leaking from the photosynthetic chain and acted as an electron bridge to transfer them back to adjacent chloroplasts. Furthermore, ZIF-8 upregulated the expression of photosynthesis-related genes, as well as genes involved in benzoxazinoid biosynthesis and secondary metabolic pathways, thereby enhancing drought resistance and photosynthetic efficiency through multiple mechanisms [148]. Poly(acrylic acid)-coated manganese oxide (Mn_3_O_4_) nanoparticles (PMOs) were applied to cotton under drought stress, resulting in a significant increase in fresh weight (74.9%) and dry weight (75.5%), alongside a reduction in ROS levels (H_2_O_2_ by 60.7% and O_2_^−^ by 67.8%). PMO treatment also enhanced CO_2_ uptake, improved net photosynthetic rate (by 160%) and transpiration rate, and increased both seed cotton yield and lint yield (by approximately 20–22%) [149].

Exposure to extreme temperatures can cause severe damage to plants, including reduced photosynthetic efficiency, shortened life cycles, and decreased yield. NMs can enhance plant thermotolerance by improving nutrient uptake, regulating osmotic balance, optimizing photosynthesis, strengthening the antioxidant defense system, and modulating phytohormone signaling pathways [150]. Application of nanocapsule–potassium (N-K) to pepper (*Capsicum annuum* L.) under high-temperature stress significantly reduced stress indicators such as malondialdehyde (MDA) and electrolyte leakage (EL), while enhancing the activities of catalase (CAT) and superoxide dismutase (SOD). N-K treatment also modulated fatty acid composition, effectively improving the growth and physiological status of pepper plants [151]. Treatment with zinc oxide nanoparticles (ZnO NPs) increased the activity of antioxidant enzymes (SOD, CAT, POD) and up-regulated the expression of related genes (e.g., *OsCu*/*ZnSODs*, *OsPRXs*, *OsCATs*). Concurrently, it down-regulated the expression of cold-responsive transcription factors (such as *OsbZIP52*, *OsMYB4*, *OsMYB30*, *OsNAC5*, *OsWRKY76*, *OsWRKY94*), significantly alleviating the inhibitory effects of chilling stress on rice (*Oryza sativa* L.) growth. Additionally, ZnO NPs restored the contents of chlorophyll a, chlorophyll b, and total chlorophyll [152].

Salt stress severely impacts crop growth, leading to yield losses and reduced quality [153]. It primarily involves osmotic stress, sodium ion (Na^+^) toxicity, and the excessive accumulation of reactive oxygen species (ROS) as a secondary stress. Current research on salt tolerance mechanisms is largely based on these aspects [154]. NMs can effectively enhance plant salt tolerance [155]. GO alleviated the inhibitory effects of salt stress (200 mM NaCl) on peanut growth, increasing plant height, root length, and biomass. Mechanistic studies revealed that GO enhanced photosynthesis (by improving net photosynthetic rate and chlorophyll content) and boosted the antioxidant system (e.g., increased activities of SOD and CAT). Additionally, GO promoted the accumulation of amino acids (such as glutamate) and phytohormones (including gibberellin and cytokinin) [142]. Tannic acid-iron (TA-Fe) NMs effectively alleviated the inhibitory effects of salt stress on rice growth. By scavenging reactive oxygen species (ROS) and protecting cell membrane integrity, TA-Fe enhanced plant stress tolerance. Moreover, as these NMs are derived from natural tannic acid, they exhibit low toxicity and high safety [156].

Heavy metal contamination is characterized by high toxicity, irreversibility, and persistence, severely impairing fundamental soil functions, reducing crop yield and quality, and posing risks to human health [157,158]. Cao et al. found that metalloid-based NMs enhanced rice biomass and reduced arsenic accumulation in plant tissues by modulating the rhizosphere microbial community, optimizing soil bacterial structure, promoting arsenic methylation, limiting arsenic uptake, and strengthening internal arsenic detoxification mechanisms. Additionally, these NMs significantly improved both rice yield and the nutritional quality of grains [159]. Two-dimensional MXene NMs (Ti_3_C_2_Tx) enhanced lead (Pb) stress tolerance in *Torreya grandis*, significantly improving growth parameters while reducing malondialdehyde (MDA) content and electrolyte leakage. MXene facilitated the transformation of Pb speciation in soil, thereby decreasing its bioavailability. It also regulated genes associated with pectin synthesis and degradation, increasing Pb immobilization within the root cell walls [160]. Both putrescine (Put) and carbon quantum dots (CQDs) have demonstrated positive effects in alleviating plant stress. When combined as Put-CQD nanoparticles and applied as a protective agent, they significantly mitigated the adverse impacts of cadmium stress on grape plants. The treatment restored leaf fresh and dry weights to near-normal levels, reduced cadmium accumulation, and enhanced overall plant stress resistance [161].

### 5.2. Defense Against Biotic Stress

Biotic stress refers to the competitive or antagonistic interactions in which biological agents such as fungi, bacteria, viruses, parasites, and insects cause damage to plants [162]. It poses serious, destructive threats to agricultural productivity and food quality, leading to substantial economic losses. NMs can trigger defense responses in plants against biotic stressors through multiple mechanisms, mitigating negative impacts and alleviating damage caused by such stress [20,163].

Foliar application of copper nanoparticles restored biomass, photosynthetic efficiency, and fatty acid composition in infected soybean plants. It reduced pathogen-induced antioxidant enzyme activity, modulated the expression of defense-related and phytohormone-related genes, and enhanced innate immune responses. This approach effectively controlled soybean sudden death syndrome caused by the fungal pathogen *Fusarium virguliforme* [164].

Fusarium wilt, caused by *Fusarium oxysporum*, can lead to yield losses of up to 50% in tomato. Application of two carbon-based nanomaterials (CNMs), carbon nanotubes (CNTs) and graphene (GP), reduced the incidence and severity of Fusarium wilt in tomato, increased fruit yield, and enhanced the content of photosynthetic pigments, ascorbic acid, and flavonoids in the leaves. Additionally, these CNMs elevated the activity of leaf antioxidant enzymes [165]. Copper oxide nanoparticles (CuO NPs) with different morphologies and surface charges exerted distinct effects on tomato plant health and disease suppression. Three types were evaluated: positively charged nanospikes (CuO (+) nanospikes), negatively charged nanospikes (CuO (−) nanospikes), and negatively charged nanosheets (CuO (−) nanosheets). Among these, negatively charged nanoparticles, particularly the nanosheets, demonstrated superior disease suppression efficacy. They significantly reduced disease progression caused by Fusarium oxysporum and increased plant biomass [166].

The pathogen *Pseudomonas syringae* pv. Tomato DC3000 (*Pst*. DC3000) causes bacterial spot disease in tomato plants. Yu et al. developed a novel nanomaterial, ZnO-TiO_2_@MSC, using corn straw as a template. This material effectively controlled tomato disease and promoted plant growth through dual mechanisms: direct antimicrobial activity and activation of the plant defense system [167]. Early blight, caused by *Alternaria solani*, can significantly reduce tomato yield. Treatment with silver nanoparticles (AgNPs) modulated plant defense mechanisms and antioxidant enzyme activity, enhancing tomato resistance to the pathogen [168].

Under biotic stress induced by Aspergillus flavus, rice exhibited significantly elevated levels of antioxidant enzymes (SOD, POD, CAT) and non-enzymatic compounds such as proline and MDA. Application of silver nanoparticles (AgNPs) effectively reduced these biomarkers, alleviated oxidative stress, and decreased aflatoxin B1 (AFB1) content in rice grains from 25.1 µg kg^−1^ to 10.6 µg kg^−1^. Additionally, AgNPs treatment improved the content of minerals, including calcium, iron, and phosphorus [169].

Foliar application of La_10_Si_6_O_27_ nanorods on rice significantly reduced sheath blight severity by 62.4% and increased grain yield by 35.4%. The treatment also enhanced the content of starch, protein, and trace elements (such as iron, copper, manganese, and molybdenum) in the grains, demonstrating an efficient, economical, environmentally friendly, and sustainable crop protection strategy. The mechanisms underlying the effects of lanthanum-based NMs on rice involve multiple pathways: they activate calmodulin (CAM) and phenylalanine ammonia-lyase (PAL), upregulate the expression of disease resistance related genes, promote lignin synthesis to strengthen cell walls against pathogen invasion, and enhance antioxidant enzyme activity to alleviate pathogen induced oxidative damage [170].

Sesame plants are often subjected to stress from *Spodoptera litura* infestation. Foliar application of chitosan nanoparticles (CNPs) activated endogenous plant defense mechanisms, including calcium ion (Ca^2+^) influx and phytohormone accumulation, while upregulating biosynthetic genes of defense metabolites such as sesamin and shanzhiside methyl ester. This treatment alleviated oxidative stress induced by insect herbivory and simultaneously enhanced the nutritional quality of the seeds [171].

RNA interference (RNAi) also demonstrates remarkable potential in the field of biotic stress resistance. This technology enables the design of specific double-stranded RNA (dsRNA) molecules that can be targeted against a wide range of fungal, bacterial, and viral diseases [172].

### 5.3. Multifactorial and Combined Stress Adaption Strategies

In actual agricultural production, stresses often interact to produce combined effects. For instance, combined heat and drought stress, along with crown and root rot diseases (CRDs) caused by *Fusarium* spp., pose severe threats to wheat growth and yield. The application of bioselenium nanoparticles (BioSeNPs) synthesized using *Lactobacillus acidophilus* ML14 not only suppressed Fusarium diseases but also enhanced wheat resilience to drought and heat stress, ultimately improving yield [173].

Heat stress combined with thrips infestation significantly inhibited soybean growth and yield, manifesting as stunted growth, leaf damage, reduced biomass, and decreased nodulation. Wang et al. investigated the effects of three carbon-based NMs (CNMs), carbon nanotubes (CNTs), graphene nanoplatelets (GNPs), and industrial carbon black (CB), on soybean growth, nodulation, and leaf health under such combined stress. While the CNMs showed limited influence on overall growth and yield, GNPs increased the chlorophyll a/b ratio, whereas CNTs exacerbated oxidative damage [174]. These findings indicate that the negative impacts of combined heat and thrips stress substantially outweigh the potential mitigating effects of CNMs on soybean growth and health.

### 5.4. Summary and Discussion

The involvement of nanotechnology can induce defense mechanisms in crops, promote growth and development, or enhance the operation of crop defense systems, significantly improving crop resilience under stress (Table 4). Current research predominantly focuses on experimental studies involving single crop species under single stress conditions, which limits practical applicability. Future research should aim to: (1) develop NMs applicable to multiple crop species or effective against multiple types of stress, in alignment with real-world agricultural conditions; (2) deepen the understanding of the specific effects of stress on plants to enable accurate and effective stress resistance; (3) investigate the mechanisms through which NMs enhance crop stress tolerance.

## 6. Challenges and Prospects for Sustainable Implementation of Nanotechnology in Agriculture

While nanotechnology holds immense potential in detection, delivery, and stress resistance, concerns remain regarding its application in agriculture and food safety. This section discusses the environmental, regulatory, and socio-economic obstacles of nanotechnology, providing a roadmap for future development.

### 6.1. Environmental Risks and Biosafety Concerns

NMs possess excellent functional properties such as small size, high reactivity, and strong permeability, but they may also pose environmental and health risks. On one hand, the long-term behavior patterns of NMs in the environment remain poorly understood. Factors such as transformation, transport, and bioavailability determine the persistence, mobility, and biological availability of NMs [175]. On the other hand, at high doses or under specific conditions, NMs can exhibit toxicity to plants [176]. For instance, CuO-NPs have been shown to induce oxidative stress in wheat, disrupt nitrogen metabolism, and lead to approximately 15% growth reduction [102]. The primary toxicity mechanisms of NMs include oxidative stress, which generates reactive oxygen species (ROS), resulting in lipid peroxidation, cell membrane damage, and impairment of critical metabolic processes [177]. Furthermore, NMs entering animals and humans through the food chain pose biosafety risks. Studies have demonstrated that various NMs can cause developmental defects, altered gene expression, and multiple toxic effects in zebrafish [178]. Additionally, reports have documented the harmful impacts of NMs on human and living systems [179,180,181]. However, the toxic and genotoxic effects of nanoparticles on plants and environmental microorganisms remain unclear, and their implications for human health are not yet fully understood.

### 6.2. Regulatory Frameworks and Standardization

The rapid advancement of nano-agriculture has outpaced the establishment of specific regulatory frameworks and standards, posing obstacles to the commercialization of NMs. In the European Union, regulations such as REACH (Registration, Evaluation, Authorisation and Restriction of Chemicals) and CLP (Classification, Labelling and Packaging) govern their use [182]. In the United States, the Environmental Protection Agency regulates nanopesticides under the Federal Insecticide, Fungicide, and Rodenticide Act (FIFRA) and the Toxic Substances Control Act (TSCA) [183]. The Food and Agriculture Organization of the United Nations/World Health Organization (FAO/WHO) provides general principles for risk analysis. However, specific standards for NMs intended for agricultural and food applications have not yet been established, and testing protocols for material characterization, toxicity, and safety remain ununified [184].

### 6.3. Technical, Economic, and Social Implementation Barriers

From a technical perspective, several challenges persist. Firstly, the impact of certain NMs on metabolic pathways after their uptake into plant cells remains unclear [185]. Secondly, functional limitations are evident, as a single nanomaterial is often applicable to only one crop or capable of performing only a single function. Thirdly, research is predominantly confined to laboratory settings, lacking validation of efficacy under real-world field conditions. Moreover, the high cost of nanoparticles places them beyond the reach of most farmers [186]. The large-scale production of NMs remains expensive, and synthesis efficiency and product yield—often overlooked in research—hinder extensive agricultural application. Additionally, social acceptance and ethical considerations are imperative, as “nano-agricultural products” may raise public safety concerns and trigger consumer resistance [187].

### 6.4. Future Perspectives

Future efforts should focus on developing non-toxic or low-toxicity, bio-friendly, biodegradable, and affordable NMs. The green synthesis of NMs using agricultural waste or plant extracts typically offers lower costs compared to engineered metal or carbon-based nanoparticles, representing an economically viable and environmentally sustainable alternative [186]. Integrating these with technologies such as the Internet of Things (IoT) and artificial intelligence (AI) will further advance precision agriculture. Multi-omics analyses should be employed to gain deeper insights into the mechanisms of NMs in plants and environmental microorganisms. A systematic evaluation of nanomaterial behavior in the soil–plant system and long-term biotoxicity effects is essential. Long-term studies on the fate and impact of NMs, combined with full life cycle assessment, will provide reliable support for understanding their toxic effects and mechanisms of action. Concurrently, regulatory frameworks for NMs should be strengthened, with urgent clarification of definitions for agricultural NMs and standardization of testing protocols and toxicity indicators. Furthermore, public education initiatives can help reduce resistance to agricultural products involving NMs.

In conclusion, realizing the potential of nanotechnology in agriculture depends not only on continuous innovation but also on proactively addressing associated risks and societal concerns, as sustainable and safe food production is of paramount importance.

## 7. Conclusions

**Figure 4:**
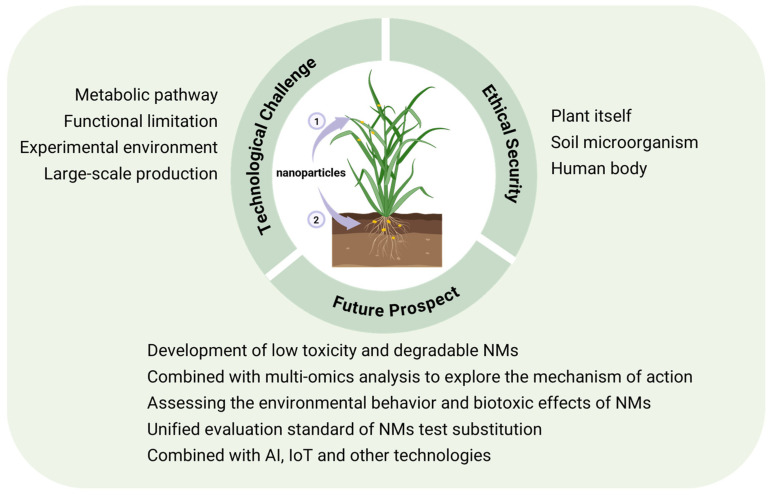
Technological challenges, ethical security and future prospect of nanotechnology in agricultural applications. (In the figure, 1 means foliar spraying, 2 means soil application.)

The application of nanotechnology has significantly enhanced agricultural sustainability. NMs are capable of penetrating plant cells and interacting with cellular membranes or organelles, thereby activating specific metabolic pathways and influencing phytohormone synthesis, as well as the expression of DNA, proteins, and enzymes [138]. This review highlights applications of nanotechnology in agriculture, including biosensors, nano-agrochemicals, and stress-resistant NMs. It elucidates the mechanisms of nano-material–plant and nano-material–soil–plant interactions across physiological, cellular, and ecological scales. Current research gaps and challenges are discussed, and future development directions are emphasized (Figure 4).

## Figures and Tables

**Table 1:** Biosensor-based detection of diverse target substances.

	Analytes	Sensing Strategy	Core Materials	Detection Parameters	Samples	Recovery (%)	References
Heavy-metal and metalloid ions							
1	Pb (II)	Electrochemical	single-walled carbon nanotubes (SWCNT) cysteine residues (Cys)	Dynamic linear range: 5.0–125.0 μg L^−1^ Sensitivity: 0.061 μA μg^−1^ L LOD: 0.69 μg L^−1^	Tap water samples	98.3	[58]
					Rainwater samples	100	
2	Pb (II)	Electrochemical	Bipedal DNA Walker, CHA, AuNPs@Zr-MOF	Linear range: 0.05–1.0 μmol L^−1^ LOD: 4.65 × 10^−6^ μmol L^−1^	Rice flour, tea, honey, vermicelli, rice	99.55–103.44 RSD < 5	[60]
3	Hg (II)	SERS	Au@Ag/COF Y-shaped DNA	Linear range: 10^−8^–10^−3^ μM LOD: 5.0 × 10^−10^ μM	River water Tap water Milk	97.9–104.0 RSD: 4.8	[46]
4	Pb (II)	DNAzymes	Platinum nanoparticle networks	Response time: 15–20 s Linear range: 10^−3^–10 μM LOD: 0.01 μM	-	-	[59]
5	Cd (II)	Colorimetric	Porous Co_3_O_4_ nanodisks with strong peroxidase-mimicking	Linear range: 0.20–10 μg L^−1^ LOD: 0.085 μg L^−1^	Tap water River water Lake water Industry water	Average: 86.9–98.3 RSD: 3.11–9.13 Mixed: 86.9–95.2 RSD: 3.11–6.23	[56]
6	Hg (II)			Linear range: 0.50–25 μg L^−1^ LOD: 0.19 μg L^−1^			
7	Pb (II)			Linear range: 0.50–20 μg L^−1^ LOD: 0.2 μg L^−1^			
8	As			Linear range: 0.40–20 μg L^−1^ LOD: 0.156 μg L^−1^			
9	Cu (II)	Visually multiplexed quantitation	TV-Chip DNA-nanoparticle	Linear range: 1.5 × 10^−3^–0.258 μM LOD: 0.001 μM	River water	-	[57]
10	Pb (II)			Linear range: 0.0015–0.258 μM LOD: 0.001 μM			
11	Hg (II)			Linear range: 0.002–0.2 μM LOD: 0.0018 μM			
Pesticide							
1	Paraoxon	Electrochemical	Carbon black/Prussian blue nanoparticle	Linear range: 2–20 μg L^−1^ LOD: 2 μg L^−1^	Standard solutions and a river water sample	90 ± 1	[63]
2	2,4-dichlorophenoxyacetic acid			Linear range: 100–600 μg L^−1^ LOD: 50 μg L^−1^		93 ± 2	
3	Atrazine			Linear range: 10–100 μg L^−1^ LOD: 10 μg L^−1^		95 ± 3	
4	Acetamiprid	Aptamer-based biosensors (aptasensors)	Fe-N-C single-atom nanozymes	LOD: 0.0169 μM	real river water samples	99.70–101.17 RSD: 4.46	[65]
5	Malathion	Electrochemiluminescence	TEMPO-MWCNTs	Linear range: 0.1–2 μg L^−1^ LOD: 0.007 μg L^−1^	Cabbage	80.56–101.80 RSD: 3.22–7.94	[64]
6	Phoxim	SERS	Fe_3_O_4_@UiO-66(Zr)@Ag nanoparticles	Linear range: 100–50,000 μg L^−1^ LOD: 41 μg L^−1^ (1.37 × 10^−7^ M)	Apple juice	97.17 ± 4.94–109.17 ± 4.77	[80]
7	Triazophos			Linear range: 100–50,000 μg L^−1^ LOD: 21 μg L^−1^ (6.70 × 10^−8^ M)		96.83 ± 0.30–116.67 ± 8.91	
8	Parathion-methyl			Linear range: 20–50,000 μg L^−1^ LOD: 3.1 μg L^−1^ (1.18 × 10^−8^ M)		96.83 ± 1.95–101.67 ± 5.68	
9	Thiram	SERS	FP/Ag/ZIF-8	LOD: 4 × 10^−5^ μM	soaking in lake water	92–102 RSD: 7.3	[81]
				LOD: 5 × 10^−3^ μM within 1 min	filtration, in peach juice		
				LOD: 0.1 ng/cm^2^	swabbing on apple peel		
10	Chloropyrifos	Colorimetric	CeGONRs	Linear range: 0.012–3.50 μg mg^−1^ LOD: 3.43 × 10^−3^ μg L^−1^	Cabbage	95–105	[82]
11	Glyphosate	Fluorescent	Sophora Japonica leaves	Linear range: 100–16,000 μg L^−1^ LOD: 8.75 μg L^−1^	Potatoes and soil	96.2–105.8 RSD < 5	[83]
Antibiotic							
1	Florfenicol	Electrochemical	CoMnN-Cs Exonuclease I	Linear range: 10^−3^–10^3^ μg L^−1^ LOD: 5.28 × 10^−4^ μg L^−1^	milk, egg, and shrimp samples	96.3–100.9	[69]
2	Chloramphenicol	Photoelectrochemical	TiO_2_@MoS_2_ spiral nanoarrays Aptamer-SH	Dynamic linear range: 0.1 × 10^−7^–1 μM LOD: 0.1 × 10^−6^ μM	treated milk	96–112	[70]
3	Ampicillin	Electrochemiluminescence	Bi_2_S_3_@Au nanoflowers	Linear range: 10^−6^–10^3^ μg L^−1^ LOD: 0.357 μg L^−1^	Scallops and fish	99.99–119.18	[84]
4	Chloramphenicol	PEC	In_2_S_3−x_Se_x_	Linear range: 5 × 10^−8^–0.01 μM LOD: 1.7 × 10^−8^ μM	-	-	[71]
5	Tetracycline	Fluorescent	Rice residue and glycine	LOD: 0.2367 μM	River, Tap, and Natural mineral water	96.65–104.28	[66]
6	Terramycin			LOD: 0.3739 μM			
7	Chlortetracycline			LOD: 0.2791 μM			
8	Oxytetracycline (OTC)	Fluorescent	2D MOF-DNA	Linear range: 0.50–5.00 μg L^−1^ LOD: 0.40 μg L^−1^	-	-	[67]
9	sulfonamides (SAs)	SPR	MoS_2_-enhanced SPR biosensor	Linear range: 0.15–6.59 μg L^−1^ LOD: 0.05 μg L^−1^	-	80.56–101.80 RSD: 3.22–7.94	[68]
10	Rifampicin	Electrochemical	CoFe_2_O_4_@CdSe	Linear range: 10^−10^–0.1 μM detection limit (4.55 × 10^−11^ μM)	Tablet sample	98.68–99.73 RSD < 1.94	[85]
					Serum sample	97.36–101.45 RSD < 3.32%	
Other pollutants							
1	Polychlorinated biphenyls (PCBs)	SP-RLS and SERS	β-cyclodextrin silver (Ag) nanoparticles	SP-RLS LOD: 0.7 × 10^−5^ μM SERS: 3 × 10^−8^ μM	Growing water and the plant *L. minor*	-	[72]
2	Bisphenol A	Electrochemical enzyme	Tyr-GDY-Chi/GC	Linear range: 0.1–3.5 μmol L^−1^ Sensitivity: 2990.8 mAcm^−2^M^−1^ LOD: 0.024 μmol L^−1^	Drinking bottles and tap water	86.4–114	[75]
3	Bisphenol A	Electrochemical	Tyr@Cu–TCPP	Linear range of 0.0035–18.9 μM LOD: 0.0012 μM	milk and plastic mineral water bottles samples	97.8–106.0	[76]
4	17β-estradiol	Electrochemical	Rhodium nanoparticles Single-layer graphene oxide Laccase	Linear range: 0.9 × 10^−7^–1.1 × 10^−5^ μM Sensitivity: 1.8 A mM^−1^ (25.7 A mM^−1^ cm^2^) LOD: 0.54 × 10^−7^ μM	Real urine samples	99.6 ± 0.8	[77]
5	Hydroquinone	Electrochemical	MoO_3_@KSC/SPE	Linear range: 5–176.8 μM LOD: 0.063 μM	River water and lake water	98.94–102.10	[73]
6	Catechol			Linear range: 5–176.8 μM LOD: 0.059 μM		97.50–100.13	
7	Catechol	Electrochemical	water-soluble graphene tyrosinase	Response time: 3 s Linear range: 0.025–11.1 μM Sensitivity: 12,600 mA cm ^−2^ M^−1^ LOD: 0.008 μM	environmental water samples	90.5–108.6	[74]
8	Ethephon	paper-based SERS	HKUST-1(Cu)/BAs/ PBSM	linear range: 10^−3^–10 mg kg^−1^ LOD: 1.39 × 10^−4^ mg kg^−1^	Banana	92	[86]
					Cucumber	96	
					Cocozelle	102	
					Synthetic urine samples	98.9 ± 0.6	
9	Zearalenone	Electrochemical	Polyacrylonitrile Graphite electrode	Linear Range: 0.005–0.03, 0.06–0.1 μM LOD: 0.00166 μM LOQ: 0.005 μM	Food simulant	-	[79]
10	Trenbolone	Electrochemiluminescence	CeO_2_/NiCo-LDH@Au	linear range: 1.0 × 10^−4^–50.0 μg L^−1^ LOD: 6.84 × 10^−5^ μg L^−1^	Lake water	96.8–101.9 RSD ≤ 3.8	[87]

LOD: limit of detection, RSD: Relative standard deviation, LOQ: limit of quantitation.

**Table 4:** Application of nanomaterials in stress resistance (↑ is increase and ↓ is decrease).

	Nanomaterial	Crop	Stress	Application Rate	Application Method	Effects	Mechanism	References
Abiotic Stress								
1	ZIF-8	Maize (*Zea maize*)	Drought stress	200 mg L^−1^	Seed priming	Photosynthetic efficiency ↑, Growth performance ↑	Repair of the electron transport chain, activation of the antioxidant system, and regulation of metabolic pathways.	[148]
2	GO	Soybeans (*Glycine max* L.)	Drought stress	100 mg L^−1^	Drench	RWC ↑, Root parameters ↑, Activity of antioxidant enzymes ↑	Expression of drought-responsive genes ↑, Levels of key phytohormones ↑	[147]
3	PMO	Cotton (*Gossypium hirsutum* L.)	Drought stress	50–800 mg L^−1^	Foliar spray	Fresh weight ↑, Dry weight ↑	ROS homeostasis ↑, Oxidative damage ↓, Photosynthesis ↑	[149]
4	Nanocapsule–potassium (N-K)	Pepper (*Capsicum annuum* L.)	Heat stress	1 μM	Foliar spray	Pepper growth ↑, photosynthetic efficiency ↑	Antioxidant defense mechanisms ↑, Osmotic balance ↑, Water retention capacity ↑, Photosynthetic ↑, Membrane lipid composition ↑	[151]
5	ZnO NPs	Rice (*Oryza sativa* L.)	Chilling stress	25, 50, 100 mg L^−1^	Foliar spray	Plant height ↑, Root length ↑, Dry biomass ↑, Leaf chlorosis ↓	Antioxidant defense system ↑, Cold-responsive transcription factors ↑, Chlorophyll biosynthesis ↑	[152]
6	Graphene oxide (GO)	Peanut (*Arachis hypogaea* L.)	Salinity stress	400 mg L^−1^	Seed priming	Peanut pod yield ↑	Accumulation of sugars and amino acids ↑, Synthesis of gibberellins ↑, auxins ↑, Cytokinins ↑, ROS accumulation ↓	[142]
7	Tannic acid-iron (TA-Fe) nanomaterial	Rice (*Oryza sativa*)	Salinity stress	25 mg L^−1^	Seed priming, hydroponic, drench	Hydroponic: Underground and aboveground lengths ↑, Fresh weight ↑ Soil-cultivated: Biomass ↑, Shoot length ↑	Neutralizes ROS: ·O_2_^−^, ·OH, H_2_O_2_	[156]
8	Metalloid NMs, including SiO_2_, hydroxyapatite, S^0^, and Se^0^	Rice (*Oryza sativa* L.)	As stress	10–100 (0.1–5.0 for Se NMs) mg kg^−1^	Soil application	Biomass ↑, Arsenic accumulation ↓	Biosynthesis pathway of abscisic acid ↑, jasmonic acid ↑, glutathione ↑, Arsenic transporter-related gene expression in roots ↓, Formation of iron plaque ↓, Arsenic detoxification ↑	[159]
9	Ti_3_C_2_Tx MXene nanosheets	*Torreya grandis*	Pb stress	100 mg kg^−1^	Soil application	Tolerance to Pb stress ↑	Converted the available form of Pb into stable forms, Cell wall pectin content ↑	[160]
10	Put-CQD NPs	Grapevine (*Vitis vinifera* L.)	Cd stress	25, 50 mg L^−1^	Foliar spray	Growth parameters ↑, photosynthesis↑	Cadmium uptake ↓, Antioxidant defense system ↑, Regulated polyamine metabolism	[161]
Biotic stress								
1	Customized Cu_3_(PO_4_)_2_ nanosheets, Customized CuO nanosheets, Commercial CuO nanoparticles	Soybean (*Glycine max*)	Soybean sudden death syndrome	250 mg L^−1^	Foliar spray	Transcription of pathogenesis-related genes ↑	Modulating nutritional status, Activating plant defense systems	[164]
2	lanthanum (La) based nanomaterials	Rice (*Oryza sativa* L.)	sheath blight (*Rhizoctonia solani*)	100 mg L^−1^	Foliar spray	Severity of sheath blight ↓, Efficacy of the commercially available pesticide ↑	Rice systemic acquired resistance ↑, Physical barrier formation ↑, Antioxidative systems ↑	[170]
3	ZnO-TiO_2_@MSC nanomaterial	Tomato (*Solanum lycopersicum*)	Bacterial leaf spot disease	2000 mg L^−1^	Foliar spray	Control of bacterial leaf spot disease ↑	Triggered plant defense mechanisms, Resistance genes ↑, Activities of key enzymes ↑	[167]
4	CuO (+) nanospikes CuO (−) nanospikes CuO (−) nanosheets	Tomato (*Solanum lycopersicum*)	*Fusarium oxysporum* f. sp. *lycopersici*	125 mg L^−1^	Foliar spray	Disease progression ↓, biomass ↑	More efficient internalization and translocation.	[166]
5	CNMs	Tomato (*Solanum lycopersicum* L.)	*Fusarium oxysporum*	CNT: 100 mg L^−1^ GP: 250 mg L^−1^	Drench	Disease incidence ↓, Yield ↑, Antioxidant capacity ↑	Directly damaged the pathogen’s cell membrane or indirectly activated the plant’s defense system.	[165]
6	AgNPs	Tomato (*Solanum lycopersicum*)	*Alternaria solani*	20 mg L^−1^	Foliar spray	Symptoms of early blight ↓, Overall health of the plants ↑	Activated defense pathways and antioxidant enzyme activity	[168]
7	AgNPs	Rice (*Oryza sativa*)	*Aspergillus flavus*	50 mg kg^−1^	Foliar spray	Alleviated oxidative stress, AFB1 content ↓	Antioxidant responses ↑, Controlled aflatoxin production, Mineral content ↑	[169]
8	Chitosan nanoparticles (CNPs)	Seeds of sesame (*Sesamum indicum* L.)	*Spodoptera litura*	100 mg L^−1^	Foliar spray	Resistance to insect pests ↑, Crop nutritional quality ↑	Activated calcium signaling pathways and phytohormone signaling pathways	[171]
Combined stress								
1	BioSeNPs	Wheat (*Triticum asetivum* L.)	Drought stress Heat stress CRDs	100 mg L^−1^	Seed priming	Plant height ↑, Root and shoot biomass ↑, Spike length ↑, 1000-grain weight ↑, Disease incidence ↓, Disease severity ↓	Disrupted fungal cell membranes and DNA, Phenolic compounds and flavonoids contributed to both antioxidant and antifungal effects	[173]
2	CNTs GNPs CB	Soybean (Genuity Roundup Ready 2 Yield Soybean, Group 2, H20R3)	Heat stress Insect (thrips) stress	1000 mg kg^−1^	Soil application	Growth parameters ↑, Final biomass ↑, Lleaf health ↑	Altered chlorophyll ratios and induced oxidative damage	[174]

## Data Availability

Data will be made available on request.

## References

[1] Ke J., Wang B., Yoshikuni Y. (2021). Microbiome Engineering: Synthetic Biology of Plant-Associated Microbiomes in Sustainable Agriculture. Trends Biotechnol..

[2] Roell M.-S., Zurbriggen M.D. (2020). The Impact of Synthetic Biology for Future Agriculture and Nutrition. Curr. Opin. Biotechnol..

[3] Lai Y.-J., Lu P.-C., Kung Y. (2025). Duckweed-Based Optical Biosensor for Herbicide Toxicity Assessment. Biosens. Bioelectron..

[4] Uddin K., Saha B.K., Wong V.N.L., Patti A.F. (2025). Organo-Mineral Fertilizer to Sustain Soil Health and Crop Yield for Reducing Environmental Impact: A Comprehensive Review. Eur. J. Agron..

[5] Vijayanand M., Issac P.K., Velayutham M., Deepak P., Thiyagarajulu N., Alam M.W., Guru A. (2025). A Sustainable Solution: Mitigating Aquatic Herbicide Contamination through Natural Product Interventions. Aquac. Int..

[6] Xiong L., Li Z., Shah F., Wang P., Yuan Q., Wu W. (2024). Biodegradable Mulch Film Enhances the Environmental Sustainability Compared with Traditional Polyethylene Film from Multidimensional Perspectives. Chem. Eng. J..

[7] Rai P.K., Kumar V., Lee S., Raza N., Kim K.-H., Ok Y.S., Tsang D.C.W. (2018). Nanoparticle-Plant Interaction: Implications in Energy, Environment, and Agriculture. Environ. Int..

[8] Yu Z., Tetard L., Zhai L., Thomas J. (2015). Supercapacitor Electrode Materials: Nanostructures from 0 to 3 Dimensions. Energy Environ. Sci..

[9] Handa M., Kalia A. (2024). Nanoparticle-Plant-Microbe Interactions Have a Role in Crop Productivity and Food Security. Rhizosphere.

[10] Tang Y., Zhao W., Zhu G., Tan Z., Huang L., Zhang P., Gao L., Rui Y. (2023). Nano-Pesticides and Fertilizers: Solutions for Global Food Security. Nanomaterials.

[11] Ding Y., Zhao W., Zhu G., Wang Q., Zhang P., Rui Y. (2023). Recent Trends in Foliar Nanofertilizers: A Review. Nanomaterials.

[12] Jiang Y., Yang J., Li M., Li Y., Zhou P., Wang Q., Sun Y., Zhu G., Wang Q., Zhang P. (2022). Effect of Silica-Based Nanomaterials on Seed Germination and Seedling Growth of Rice (*Oryza sativa* L.). Nanomaterials.

[13] Ullah S., Adeel M., Zain M., Rizwan M., Irshad M.K., Jilani G., Hameed A., Khan A., Arshad M., Raza A. (2020). Physiological and Biochemical Response of Wheat (*Triticum aestivum*) to TiO_2_ Nanoparticles in Phosphorous Amended Soil: A Full Life Cycle Study. J. Environ. Manag..

[14] Li M., Zhang P., Guo Z., Zhao W., Li Y., Yi T., Cao W., Gao L., Tian C.F., Chen Q. (2024). Dynamic Transformation of Nano-MoS_2_ in a Soil–Plant System Empowers Its Multifunctionality on Soybean Growth. Environ. Sci. Technol..

[15] Guo H., White J.C., Wang Z., Xing B. (2018). Nano-Enabled Fertilizers to Control the Release and Use Efficiency of Nutrients. Curr. Opin. Environ. Sci. Health.

[16] Shukla P. (2019). Synthetic Biology Perspectives of Microbial Enzymes and Their Innovative Applications. Indian J. Microbiol..

[17] Singh R., Umapathi A., Patel G., Patra C., Malik U., Bhargava S.K., Daima H.K. (2023). Nanozyme-Based Pollutant Sensing and Environmental Treatment: Trends, Challenges, and Perspectives. Sci. Total Environ..

[18] Kumar A., Choudhary A., Kaur H., Mehta S., Husen A. (2021). Smart Nanomaterial and Nanocomposite with Advanced Agrochemical Activities. Nanoscale Res. Lett..

[19] Wahab A., Muhammad M., Ullah S., Abdi G., Shah G.M., Zaman W., Ayaz A. (2024). Agriculture and Environmental Management through Nanotechnology: Eco-Friendly Nanomaterial Synthesis for Soil-Plant Systems, Food Safety, and Sustainability. Sci. Total Environ..

[20] Omran B.A., Baek K.-H. (2022). Control of Phytopathogens Using Sustainable Biogenic Nanomaterials: Recent Perspectives, Ecological Safety, and Challenging Gaps. J. Clean. Prod..

[21] Law S.S.Y., Miyamoto T., Numata K. (2023). Organelle-Targeted Gene Delivery in Plants by Nanomaterials. Chem. Commun..

[22] Goyal V., Rani D., Ritika, Mehrotra S., Deng C., Wang Y. (2023). Unlocking the Potential of Nano-Enabled Precision Agriculture for Efficient and Sustainable Farming. Plants.

[23] Hussain M., Zahra N., Lang T., Zain M., Raza M., Shakoor N., Adeel M., Zhou H. (2023). Integrating Nanotechnology with Plant Microbiome for Next-Generation Crop Health. Plant Physiol. Biochem..

[24] Azeem I., Adeel M., Ahmad M.A., Shakoor N., Jiangcuo G.D., Azeem K., Ishfaq M., Shakoor A., Ayaz M., Xu M. (2021). Uptake and Accumulation of Nano/Microplastics in Plants: A Critical Review. Nanomaterials.

[25] Yan X., Chen S., Pan Z., Zhao W., Rui Y., Zhao L. (2023). AgNPs-Triggered Seed Metabolic and Transcriptional Reprogramming Enhanced Rice Salt Tolerance and Blast Resistance. ACS Nano.

[26] Zhao W., Wu Z., Amde M., Zhu G., Wei Y., Zhou P., Zhang Q., Song M., Tan Z., Zhang P. (2023). Nanoenabled Enhancement of Plant Tolerance to Heat and Drought Stress on Molecular Response. J. Agric. Food Chem..

[27] Wang M., Liu Z., Yang F., Bu Q., Song X., Yuan S. (2025). Multimodal Fusion-Driven Pesticide Residue Detection: Principles, Applications, and Emerging Trends. Nanomaterials.

[28] Sutariya K., Menaka C., Shahid M., Kashyap S., Choudhary D., Padmanabhan S. (2025). Leveraging AI in Cloud Computing to Enhance Nano Grid Operations and Performance in Agriculture. Sustain. Comput. Inform. Syst..

[29] D C.S., Devarajan Y. (2025). Investigation of Emerging Technologies in Agriculture: An in-Depth Look at Smart Farming, Nano-Agriculture, AI, and Big Data. J. Biosyst. Eng..

[30] Singh H., Sharma A., Bhardwaj S.K., Arya S.K., Bhardwaj N., Khatri M. (2021). Recent Advances in the Applications of Nano-Agrochemicals for Sustainable Agricultural Development. Environ. Sci. Process. Impacts.

[31] Zhai Y., Hellmann R., Campos A., Findling N., Mayanna S., Wirth R., Schreiber A., Cabié M., Zeng Q., Liu S. (2021). Fertilizer Derived from Alkaline Hydrothermal Alteration of K-Feldspar: A Micrometer to Nanometer-Scale Investigation of K in Secondary Reaction Products and the Feldspar Interface. Appl. Geochem..

[32] Liang M., Liu Y., Lu S., Wang Y., Gao C., Fan K., Liu H. (2024). Two-Dimensional Conductive MOFs toward Electrochemical Sensors for Environmental Pollutants. TrAC Trends Anal. Chem..

[33] Kaur I., Batra V., Kumar Reddy Bogireddy N., Torres Landa S.D., Agarwal V. (2023). Detection of Organic Pollutants, Food Additives and Antibiotics Using Sustainable Carbon Dots. Food Chem..

[34] Shelar A., Salve S., Shende H., Mehta D., Chaskar M., Nile S.H., Patil R. (2024). Recent Advances on Highly Sensitive Plasmonic Nanomaterial Enabled Sensors for the Detection of Agrotoxins: Current Progress and Future Perspective. Comput. Electron. Agric..

[35] Azzouz A., Kumar V., Hejji L., Kim K.-H. (2023). Advancements in Nanomaterial-Based Aptasensors for the Detection of Emerging Organic Pollutants in Environmental and Biological Samples. Biotechnol. Adv..

[36] Vikesland P.J. (2018). Nanosensors for Water Quality Monitoring. Nat. Nanotechnol..

[37] Joshi A., Kim K.-H. (2020). Recent Advances in Nanomaterial-Based Electrochemical Detection of Antibiotics: Challenges and Future Perspectives. Biosens. Bioelectron..

[38] Das A., Uppaluri R.V.S., Mitra S. (2025). A Review on Waste Derived Carbon Nanozyme: An Emerging Catalytic Material for Monitoring and Degrading Environmental Pollutants. Chem. Eng. J..

[39] Zhang W., Asiri A.M., Liu D., Du D., Lin Y. (2014). Nanomaterial-Based Biosensors for Environmental and Biological Monitoring of Organophosphorus Pesticides and Nerve Agents. TrAC Trends Anal. Chem..

[40] Tian Y., Liu J., Qiao J., Ge F., Yang Y., Zhang Q. (2025). Advancements in Electrochemical Sensing Technology for Heavy Metal Ions Detection. Food Chem. X.

[41] Bist D.R., Chapagaee P., Kunwar A., Pant B.D., Khatri L., Mandal A. (2025). Nanotechnology in Agriculture: A Review of Innovations in Crop Protection and Food Security. Adv. Agric..

[42] Raval J.B., Mehta V.N., Singhal R.K., Basu H., Jha S., Kailasa S.K. (2023). Insights of Nanomaterials Integrated Analytical Approaches for Detection of Plant Hormones in Agricultural and Environmental Samples. Trends Environ. Anal. Chem..

[43] Seddaoui N., Arduini F. (2025). Recent Advances in Wearable and Implantable Electrochemical (Bio)Sensors for Plant Health Monitoring. TrAC Trends Anal. Chem..

[44] Sahragard A., Varanusupakul P., Miró M. (2023). Nanomaterial Decorated Electrodes in Flow-through Electrochemical Sensing of Environmental Pollutants: A Critical Review. Trends Environ. Anal. Chem..

[45] Rasheed T., Hassan A.A., Kausar F., Sher F., Bilal M., Iqbal H.M.N. (2020). Carbon Nanotubes Assisted Analytical Detection–Sensing/Delivery Cues for Environmental and Biomedical Monitoring. TrAC Trends Anal. Chem..

[46] Li Y., Zhou N., Yan J., Cui K., Chu Q., Chen X., Luo X., Deng X. (2024). A Dual-Signaling Surface-Enhanced Raman Spectroscopy Ratiometric Strategy for Ultrasensitive Hg^2+^ Detection Based on Au@Ag/COF Composites. Food Chem..

[47] Amirinezhadfard E., Niazi Tabar A., Bashir M., Yang W.-C. (2025). Plant Osmosensors in Next-Generation Smart Agriculture: From Innovation to Application. Ind. Crops Prod..

[48] Dar F.A., Qazi G., Pirzadah T.B., Hakeem K.R., Pirzadah T.B. (2020). Nano-Biosensors: NextGen Diagnostic Tools in Agriculture. Nanobiotechnology in Agriculture: An Approach Towards Sustainability.

[49] Tong S.W., Goh W.P., Jiang C. (2023). Review—Recent Advances in Nanosensors for Precision Agriculture. J. Electrochem. Soc..

[50] Mirres A.C.D.M., Silva B.E.P.D.M.D., Tessaro L., Galvan D., Andrade J.C.D., Aquino A., Joshi N., Conte-Junior C.A. (2022). Recent Advances in Nanomaterial-Based Biosensors for Pesticide Detection in Foods. Biosensors.

[51] Yang Y., Liu S., Shi P., Zhao G. (2022). A Highly Sensitive and Selective Label-free Electrochemical Biosensor with a Wide Range of Applications for Bisphenol a Detection. Electroanalysis.

[52] Gaviria M.I., Barrientos K., Arango J.P., Cano J.B., Peñuela G.A. (2022). Highly Sensitive Fluorescent Biosensor Based on Acetylcholinesterase and Carbon Dots–Graphene Oxide Quenching Test for Analytical and Commercial Organophosphate Pesticide Detection. Front. Environ. Sci..

[53] Ullah N., Mansha M., Khan I., Qurashi A. (2018). Nanomaterial-Based Optical Chemical Sensors for the Detection of Heavy Metals in Water: Recent Advances and Challenges. TrAC Trends Anal. Chem..

[54] Huang D., Ma H., Wang J., Du Y., Li R. (2024). Mof-Mediated Paper-Based (Bio)Sensors for Detecting of Food and Environmental Pollutants: Preparation Strategies and Emerging Applications. Microchem. J..

[55] Abdelhamid H.N., Georgouvelas D., Edlund U., Mathew A.P. (2022). CelloZIFPaper: Cellulose-ZIF Hybrid Paper for Heavy Metal Removal and Electrochemical Sensing. Chem. Eng. J..

[56] Zou W., Tang Y., Zeng H., Wang C., Wu Y. (2021). Porous Co_3_O_4_ Nanodisks as Robust Peroxidase Mimetics in an Ultrasensitive Colorimetric Sensor for the Rapid Detection of Multiple Heavy Metal Residues in Environmental Water Samples. J. Hazard. Mater..

[57] Liu X., Wang Y., Song Y. (2018). Visually Multiplexed Quantitation of Heavy Metal Ions in Water Using Volumetric Bar-Chart Chip. Biosens. Bioelectron..

[58] Ramírez M.L., Tettamanti C.S., Gutierrez F.A., Gonzalez-Domínguez J.M., Ansón-Casaos A., Hernández-Ferrer J., Martínez M.T., Rivas G.A., Rodríguez M.C. (2018). Cysteine Functionalized Bio-Nanomaterial for the Affinity Sensing of Pb(II) as an Indicator of Environmental Damage. Microchem. J..

[59] Skotadis E., Tsekenis G., Chatzipetrou M., Patsiouras L., Madianos L., Bousoulas P., Zergioti I., Tsoukalas D. (2017). Heavy Metal Ion Detection Using DNAzyme-Modified Platinum Nanoparticle Networks. Sens. Actuators B Chem..

[60] Liang R., Dong J., Li J., Jin H., Wei M., Bai T., Ren W., Xu Y., He B., Suo Z. (2024). DNAzyme-Driven Bipedal DNA Walker and Catalytic Hairpin Assembly Multistage Signal Amplified Electrochemical Biosensor Based on Porous AuNPs@Zr-MOF for Detection of Pb^2+^. Food Chem..

[61] Fang L., Liao X., Jia B., Shi L., Kang L., Zhou L., Kong W. (2020). Recent Progress in Immunosensors for Pesticides. Biosens. Bioelectron..

[62] Bhandari G., Atreya K., Scheepers P.T.J., Geissen V. (2020). Concentration and Distribution of Pesticide Residues in Soil: Non-Dietary Human Health Risk Assessment. Chemosphere.

[63] Arduini F., Cinti S., Caratelli V., Amendola L., Palleschi G., Moscone D. (2019). Origami Multiple Paper-Based Electrochemical Biosensors for Pesticide Detection. Biosens. Bioelectron..

[64] Li H., Zhong D., Zhao G., Yang Y., Yang Z., Wang C. (2024). Multimodal Generation of Luminol Electrochemiluminescence through TEMPO Functionalized Carbon Nanotubes. Chem. Eng. J..

[65] Yu H., Wang C., Xiong X., Dai B., Wang Y., Feng Z., Luo H., Zhu J., Shen G., Deng Y. (2023). Development of Fe-N-C Single-Atom Nanozymes Assisted Aptasensor for the Detection of Acetamiprid in Water Samples. Microchem. J..

[66] Qi H., Teng M., Liu S., Li J., Yu H., Teng C., Huang Z., Liu H., Shao Q., Umar A. (2019). Biomass-Derived Nitrogen-Doped Carbon Quantum Dots: Highly Selective Fluorescent Probe for Detecting Fe^3+^ Ions and Tetracyclines. J. Colloid Interface Sci..

[67] Tan B., Wang D., Cai Z., Quan X., Zhao H. (2020). Extending Suitability of Physisorption Strategy in Fluorescent Platforms Design: Surface Passivation and Covalent Linkage on MOF Nanosheets with Enhanced OTC Detection Sensitivity. Sens. Actuators B Chem..

[68] Tan J., Chen Y., He J., Occhipinti L.G., Wang Z., Zhou X. (2023). Two-Dimensional Material-Enhanced Surface Plasmon Resonance for Antibiotic Sensing. J. Hazard. Mater..

[69] Wang M., He B., Xie L., Cao X., Ren W., Suo Z., Xu Y., Wei M., Jin H. (2024). MOF-Derived Mn, N Co-Doped Co-C Nanomaterials and Exo I-Driven Dual Signal Amplification for Sensitive Detection of Florfenicol Using an Electrochemical Aptasensor. Chem. Eng. J..

[70] Sun Y., Ma C., Wu M., Jia C., Feng S., Zhao J., Liang L. (2022). Sensitivity of Photoelctrocehmical Aptasensor Using Spiral Nanorods for Detecting Antiobiotic Levels in Experimental and Real Samples. Talanta.

[71] Liu Y., Ai S., Yuan R., Liu H. (2022). Defective Se-Doped In_2_S_3_ Nanomaterial-Based Photoelectrochemical Biosensor for the Ultrasensitive Detection of Chloramphenicol. Sens. Actuators B Chem..

[72] Cui J., Chen S., Wang Y., Ji Z., Lu W., Zhu Y., Ma Y., Chen F., Zhang G. (2023). One-Pot Preparation of Supramolecularly Functionalized Silver Nanoparticles for Surface Plasmon Resonance Based Dual-Modal Sensing of Phytotoxic Polychlorinated Biphenyl. Anal. Methods.

[73] Ganesan S., Sivam S., Elancheziyan M., Senthilkumar S., Ramakrishan S.G., Soundappan T., Ponnusamy V.K. (2022). Novel Delipidated Chicken Feather Waste-Derived Carbon-Based Molybdenum Oxide Nanocomposite as Efficient Electrocatalyst for Rapid Detection of Hydroquinone and Catechol in Environmental Waters. Environ. Pollut..

[74] Lu X., Wang X., Jin J., Zhang Q., Chen J. (2014). Electrochemical Biosensing Platform Based on Amino Acid Ionic Liquid Functionalized Graphene for Ultrasensitive Biosensing Applications. Biosens. Bioelectron..

[75] Wu L., Gao J., Lu X., Huang C., Dhanjai, Chen J. (2020). Graphdiyne: A New Promising Member of 2D All-Carbon Nanomaterial as Robust Electrochemical Enzyme Biosensor Platform. Carbon.

[76] Ma J., Yuan J., Xu Y., Jiang Y., Bai W., Zheng J. (2022). Ultrasensitive Electrochemical Determination of Bisphenol a in Food Samples Based on a Strategy for Activity Enhancement of Enzyme: Layer-by-Layer Self-Assembly of Tyrosinase between Two-Dimensional Porphyrin Metal–Organic Framework Nanofilms. Chem. Eng. J..

[77] Povedano E., Cincotto F.H., Parrado C., Díez P., Sánchez A., Canevari T.C., Machado S.A.S., Pingarrón J.M., Villalonga R. (2017). Decoration of Reduced Graphene Oxide with Rhodium Nanoparticles for the Design of a Sensitive Electrochemical Enzyme Biosensor for 17β-Estradiol. Biosens. Bioelectron..

[78] Sohrabi H., Majidi M.R., Arbabzadeh O., Khaaki P., Pourmohammad S., Khataee A., Orooji Y. (2022). Recent Advances in the Highly Sensitive Determination of Zearalenone Residues in Water and Environmental Resources with Electrochemical Biosensors. Environ. Res..

[79] Moradi S., Azizi-Lalabadi M., Bagheri V., Sadeghi E. (2020). Fabrication of Electrospun Sensor Based on a Synthesized Component Doped into PAN (Polyacrylonitrile) Nanofibers for Electrochemical Detection of Zearalenone Mycotoxin in Foods Simulant. Sens. Bio-Sens. Res..

[80] Lv M., Pu H., Sun D.-W. (2023). Preparation of Fe_3_O_4_@UiO-66(Zr)@ag NPs Core-Shell-Satellite Structured SERS Substrate for Trace Detection of Organophosphorus Pesticides Residues. Spectrochim. Acta Part A Mol. Biomol. Spectrosc..

[81] Xu F., Shang W., Xuan M., Ma G., Ben Z. (2022). Layered Filter Paper-Silver Nanoparticle-ZIF-8 Composite for Efficient Multi-Mode Enrichment and Sensitive SERS Detection of Thiram. Chemosphere.

[82] Lin L., Ma H., Yang C., Chen W., Zeng S., Hu Y. (2020). A Colorimetric Sensing Platform Based on Self-Assembled 3D Porous CeGONR Nanozymes for Label-Free Visual Detection of Organophosphate Pesticides. Mater. Adv..

[83] Hou J., Wang X., Lan S., Zhang C., Hou C., He Q., Huo D. (2020). A Turn-on Fluorescent Sensor Based on Carbon Dots from *Sophora japonica* Leaves for the Detection of Glyphosate. Anal. Methods.

[84] Zhai H., Wang Y., Geng L., Guo Q., Zhang Y., Yang Q., Sun X., Guo Y., Zhang Y. (2023). Bipotential-Resolved Electrochemiluminescence Biosensor Based on Bi_2_S_3_@au Nanoflowers for Simultaneous Detection of Cd(II) and Ampicillin in Aquatic Products. Food Chem..

[85] Asadpour-Zeynali K., Mollarasouli F. (2017). Novel Electrochemical Biosensor Based on PVP Capped CoFe_2_O_4_@CdSe Core-Shell Nanoparticles Modified Electrode for Ultra-Trace Level Determination of Rifampicin by Square Wave Adsorptive Stripping Voltammetry. Biosens. Bioelectron..

[86] Xiu W., Zhao P., Pan Y., Wang X., Zhang L., Ge S., Yu J. (2023). Flexible SERS Strip Based on HKUST-1(Cu)/Biomimetic Antibodies Composite Multilayer for Trace Determination of Ethephon. Anal. Chim. Acta.

[87] Hao Y., Wang H., Wang C., Zhang B., Ma H., Wu D., Ren X., Li Y., Wei Q. (2025). Polyaniline-Derived Lotus-Pod Nano-Hybrid Incorporated Luminescent N-CDs Combined with Novel Co-Reaction Accelerator for Ultra-Sensitive Electrochemiluminescence Detection of Trenbolone. Microchem. J..

[88] Safdar M., Kim W., Park S., Gwon Y., Kim Y.-O., Kim J. (2022). Engineering Plants with Carbon Nanotubes: A Sustainable Agriculture Approach. J. Nanobiotechnol..

[89] Smith A.M., Gilbertson L.M. (2018). Rational Ligand Design to Improve Agrochemical Delivery Efficiency and Advance Agriculture Sustainability. ACS Sustain. Chem. Eng..

[90] Li C., Yan B. (2020). Opportunities and Challenges of Phyto-Nanotechnology. Environ. Sci. Nano.

[91] Ahmad M.A., Adeel M., Shakoor N., Ali I., Ishfaq M., Haider F.U., Deng X. (2023). Unraveling the Roles of Modified Nanomaterials in Nano Enabled Agriculture. Plant Physiol. Biochem..

[92] Kekeli M.A., Wang Q., Rui Y. (2025). The Role of Nano-Fertilizers in Sustainable Agriculture: Boosting Crop Yields and Enhancing Quality. Plants.

[93] Sun Y., Zhu G., Zhao W., Jiang Y., Wang Q., Wang Q., Rui Y., Zhang P., Gao L. (2022). Engineered Nanomaterials for Improving the Nutritional Quality of Agricultural Products: A Review. Nanomaterials.

[94] Muñoz-Bautista J.M., Bernal-Mercado A.T., Martínez-Cruz O., Burgos-Hernández A., López-Zavala A.A., Ruiz-Cruz S., Ornelas-Paz J.D.J., Borboa-Flores J., Ramos-Enríquez J.R., Del-Toro-Sánchez C.L. (2025). Environmental and Health Impacts of Pesticides and Nanotechnology as an Alternative in Agriculture. Agronomy.

[95] Pagano M., Lunetta E., Belli F., Mocarli G., Cocozza C., Cacciotti I. (2025). Advancements in Agricultural Nanotechnology: An Updated Review. Plants.

[96] Santhosh R., Vignesh M., Vikraman S.U., Selvakumar S. (2025). Revolutionizing Weed Management with Nanotechnology: A Review. Plant Sci. Today.

[97] Carmona E.R., Rojo C., Vergara Carmona V. (2024). Nanomaterial-Based Biofortification: Potential Benefits and Impacts of Crops. J. Agric. Food Chem..

[98] Ma G., Zou Y., Wang S., Guo X., Li Z., Li H., Li X., Pan X. (2025). Advancing Sustainable Agriculture with Mesoporous Nanomaterials for Smart Pesticide Delivery. J. Agric. Food Chem..

[99] Ji Y., Ma S., Lv S., Wang Y., Lü S., Liu M. (2021). Nanomaterials for Targeted Delivery of Agrochemicals by an All-in-One Combination Strategy and Deep Learning. ACS Appl. Mater. Interfaces.

[100] Toksha B., Sonawale V.A.M., Vanarase A., Bornare D., Tonde S., Hazra C., Kundu D., Satdive A., Tayde S., Chatterjee A. (2021). Nanofertilizers: A Review on Synthesis and Impact of Their Use on Crop Yield and Environment. Environ. Technol. Innov..

[101] Elsayed M.E.A., Ayoub H.A., Helal M.I.D., Sang W., Shen Z., Abdelhafeez I.A. (2025). Nanotechnology-Enabled Soil Management for Sustainable Agriculture: Interactions, Challenges, and Prospects. Environ. Sci. Nano.

[102] Mallik T., Ghosh S., Ekka D. (2025). N-Doped Graphene Oxide Nanomaterial: Synthesis and Application as Controlled-Release of Urea for Advancement in Modern Agriculture. Discov. Nano.

[103] Zhang T., Chen X., Gu H., Chen H., Huang K., Wang J., Xu H., Zhang Y., Li W. (2025). Preparation and Application of Core–Shell Nanocarbon-Based Slow-Release Foliar Fertilizer. Nanomaterials.

[104] Tan J., Zhao S., Chen J., Pan X., Li C., Liu Y., Wu C., Li W., Zheng M. (2023). Preparation of Nitrogen-Doped Carbon Dots and Their Enhancement on Lettuce Yield and Quality. J. Mater. Chem. B.

[105] López-Rayo S., Imran A., Bruun Hansen H.C., Schjoerring J.K., Magid J. (2017). Layered Double Hydroxides: Potential Release-on-Demand Fertilizers for Plant Zinc Nutrition. J. Agric. Food Chem..

[106] Marmiroli M., Pagano L., Rossi R., De La Torre-Roche R., Lepore G.O., Ruotolo R., Gariani G., Bonanni V., Pollastri S., Puri A. (2021). Copper Oxide Nanomaterial Fate in Plant Tissue: Nanoscale Impacts on Reproductive Tissues. Environ. Sci. Technol..

[107] He E., Li X., Xu X., Fu Z., Romero-Freire A., Qiu H. (2024). Distinct Accumulation Patterns, Translocation Efficiencies, and Impacts of Nano-Fertilizer and Nano-Pesticide in Wheat through Foliar versus Soil Application. J. Hazard. Mater..

[108] Yang M., Gao H., Zou H., Sun D. (2025). Siloxane and Lauric Acid Copper Dual Modification Improves Hydrophobicity and Elasticity of All Biopolymer Coated Fertilizers with Enhanced Nitrogen Release Abilities. Ind. Crops Prod..

[109] Zhang S., Gao N., Shen T., Yang Y., Gao B., Li Y.C., Wan Y. (2019). One-Step Synthesis of Superhydrophobic and Multifunctional Nano Copper-Modified Bio-Polyurethane for Controlled-Release Fertilizers with “Multilayer Air Shields”: New Insight of Improvement Mechanism. J. Mater. Chem. A.

[110] Cao X., Wang Z. (2022). Application of Nano-Agricultural Technology for Biotic Stress Management: Mechanisms, Optimization, and Future Perspectives. Environ. Sci. Nano.

[111] Ma L., Yu M., Ma Y., Gao L., Pan S., Li X., Wu X., Xu Y., Pang S., Wang P. (2023). Ascendancy of Pyraclostrobin Nanocapsule Formulation against *Rhizoctonia solani*: From a Perspective of Fungus. Pestic. Biochem. Physiol..

[112] Wang C., Zhu H., Li N., Wu Q., Wang S., Xu B., Wang Y., Cui H. (2021). Dinotefuran Nano-Pesticide with Enhanced Valid Duration and Controlled Release Properties Based on a Layered Double Hydroxide Nano-Carrier. Environ. Sci. Nano.

[113] Yan S., Gu N., Peng M., Jiang Q., Liu E., Li Z., Yin M., Shen J., Du X., Dong M. (2022). A Preparation Method of Nano-Pesticide Improves the Selective Toxicity toward Natural Enemies. Nanomaterials.

[114] Zhang Y., Meng J., Qian X., Chao Z., Zong X., Jiang Q., An S., Shen J., Yan S. (2024). Nanocarrier-Delivered Gene Silence in Juvenile Hormone Signaling Pathway: Conserved Dual Targets for Efficient Aphid Control. Entomol. Gen..

[115] Santana I., Wu H., Hu P., Giraldo J.P. (2020). Targeted Delivery of Nanomaterials with Chemical Cargoes in Plants Enabled by a Biorecognition Motif. Nat. Commun..

[116] Zuo J., Yan H., Lan R., Cai J., Lin Y., Wu W., Chen H., Hao L., Zhou X., Zhou H. (2024). An Enzyme-Responsive Core-Double Shell Structured Nano Pesticide Delivery System for Improving the UV Stability of Emamectin Benzoate (EB). Ind. Crops Prod..

[117] Song S., Jiang X., Shen H., Wu W., Shi Q., Wan M., Zhang J., Mo H., Shen J. (2021). MXene (Ti_3_C_2_) Based Pesticide Delivery System for Sustained Release and Enhanced Pest Control. ACS Appl. Bio Mater..

[118] Hao L., Lin G., Lian J., Chen L., Zhou H., Chen H., Xu H., Zhou X. (2020). Carboxymethyl Cellulose Capsulated Zein as Pesticide Nano-Delivery System for Improving Adhesion and Anti-UV Properties. Carbohydr. Polym..

[119] Liu T., Qian K., Fan J., Qin Z., Yi L., Wang J., Dou W. (2024). Nano-Based Smart Pesticide Formulations Utilizing Starch Nanocrystals: Achieving High Stability and Extended Effective Duration. Ind. Crops Prod..

[120] Yin Y., Yang M., Xi J., Cai W., Yi Y., He G., Dai Y., Zhou T., Jiang M. (2020). A Sodium Alginate-Based Nano-Pesticide Delivery System for Enhanced In Vitro Photostability and Insecticidal Efficacy of Phloxine B. Carbohydr. Polym..

[121] Han J., Liu G., Hou Y., Zhou A., Zhou J., Chen G., Lv H., Zhang Y., Lv J., Chen J. (2024). Fabrication of Novel Porous Nano-Pesticides by Modifying MPN onto Cu-TCPP MOFs to Enhance Bactericidal Efficacy and Modulate Its Bioavailability. Nano Lett..

[122] Huang W., Wang M., Hu Z., Yang T., Pei H., Zhang F. (2023). Multifunctional Metal-Organic Framework with pH-Response for Co-Delivery of Prochloraz and siRNA to Synergistic Control Pathogenic Fungi. Colloids Surf. A Physicochem. Eng. Asp..

[123] Liu D., Chen H., Mao Z., Wu M., Hua J., Hua Y., Feng C., He Z., Moffett P., Zhang K. (2025). Design of a Nano-Pesticide Combining Luvangetin and RNAi for High-Efficiency Green Management of Plant Pathogens. Chem. Eng. J..

[124] Malandrakis A.A., Kavroulakis N., Chrysikopoulos C.V. (2020). Synergy between Cu-NPs and Fungicides against *Botrytis cinerea*. Sci. Total Environ..

[125] Zhu Q., Wang B., Tan J., Liu T., Li L., Liu Y.-G. (2020). Plant Synthetic Metabolic Engineering for Enhancing Crop Nutritional Quality. Plant Commun..

[126] Zhang Q., Luo H., Xing P., Gu Q., Yi W., Yu X., Zuo C., Tang X. (2024). Responses of Hybrid Rice (*Oryza sativa* L.) Plants to Different Application Modes of Nanosized Selenium. Plants.

[127] Ali A., Mashwani Z.-R., Raja N.I., Mohammad S., Ahmad M.S., Luna-Arias J.P. (2024). Exposure of *Caralluma tuberculata* to Biogenic Selenium Nanoparticles as In Vitro Rooting Agent: Stimulates Morpho-Physiological and Antioxidant Defense System. PLoS ONE.

[128] Wang Y., Feng L.-J., Sun X.-D., Zhang M., Duan J.-L., Xiao F., Lin Y., Zhu F.-P., Kong X.-P., Ding Z. (2023). Incorporation of Selenium Derived from Nanoparticles into Plant Proteins In Vivo. ACS Nano.

[129] Guha T., Gopal G., Das H., Mukherjee A., Kundu R. (2021). Nanopriming with Zero-Valent Iron Synthesized Using Pomegranate Peel Waste: A “Green” Approach for Yield Enhancement in *Oryza sativa* L. Cv. Gonindobhog. Plant Physiol. Biochem..

[130] Lahiani M.H., Chen J., Irin F., Puretzky A.A., Green M.J., Khodakovskaya M.V. (2015). Interaction of Carbon Nanohorns with Plants: Uptake and Biological Effects. Carbon.

[131] Mirza F.S., Aftab Z.-H., Ali M.D., Aftab A., Anjum T., Rafiq H., Li G. (2022). Green Synthesis and Application of GO Nanoparticles to Augment Growth Parameters and Yield in Mungbean (*Vigna radiata* L.). Front. Plant Sci..

[132] Zhang P., Guo Z., Luo W., Monikh F.A., Xie C., Valsami-Jones E., Lynch I., Zhang Z. (2020). Graphene Oxide-Induced pH Alteration, Iron Overload, and Subsequent Oxidative Damage in Rice (*Oryza sativa* L.): A New Mechanism of Nanomaterial Phytotoxicity. Environ. Sci. Technol..

[133] Park S., Choi K.S., Kim S., Gwon Y., Kim J. (2020). Graphene Oxide-Assisted Promotion of Plant Growth and Stability. Nanomaterials.

[134] Qin Q., Wang Q., Chen Y., Tang Y., Ding Y., Rui Y. (2025). Enhancing Crop Yields and Quality of Agricultural Products: Research Progress in Nanofertilizer Applications. Environ. Sci. Nano.

[135] Sodhi G.K., Wijesekara T., Kumawat K.C., Adhikari P., Joshi K., Singh S., Farda B., Djebaili R., Sabbi E., Ramila F. (2025). Nanomaterials–Plants–Microbes Interaction: Plant Growth Promotion and Stress Mitigation. Front. Microbiol..

[136] Raimondi G., Maucieri C., Toffanin A., Renella G., Borin M. (2021). Smart Fertilizers: What Should We Mean and Where Should We Go?. Ital. J. Agron..

[137] Zhang W., Xiang Y., Fan H., Wang L., Xie Y., Zhao G., Liu Y. (2020). Biodegradable Urea–Formaldehyde/PBS and Its Ternary Nanocomposite Prepared by a Novel and Scalable Reactive Extrusion Process for Slow-Release Applications in Agriculture. J. Agric. Food Chem..

[138] Faseela P., Joel J.M., Johnson R., Janeeshma E., Sameena P.P., Sen A., Puthur J.T. (2024). Paradoxical Effects of Nanomaterials on Plants: Phytohormonal Perspective Exposes Hidden Risks amidst Potential Benefits. Plant Physiol. Biochem..

[139] Zhang P., Jiang Y., Schwab F., Monikh F.A., Grillo R., White J.C., Guo Z., Lynch I. (2024). Strategies for Enhancing Plant Immunity and Resilience Using Nanomaterials for Sustainable Agriculture. Environ. Sci. Technol..

[140] Wu Y., Wang Y., Liu X., Zhang C. (2024). Unveiling Key Mechanisms: Transcriptomic Meta-Analysis of Diverse Nanomaterial Applications Addressing Biotic and Abiotic Stresses in *Arabidopsis thaliana*. Sci. Total Environ..

[141] Ochoa L., Shrivastava M., Srivastava S., Cota-Ruiz K., Zhao L., White J.C., Hernandez-Viezcas J.A., Gardea-Torresdey J.L. (2025). Nanomaterials for Managing Abiotic and Biotic Stress in the Soil–Plant System for Sustainable Agriculture. Environ. Sci. Nano.

[142] Yan N., Cao J., Wang J., Zou X., Yu X., Zhang X., Si T. (2024). Seed Priming with Graphene Oxide Improves Salinity Tolerance and Increases Productivity of Peanut through Modulating Multiple Physiological Processes. J. Nanobiotechnol..

[143] Jia Y., Kang L., Wu Y., Zhou C., Li D., Li J., Pan C. (2023). Review on Pesticide Abiotic Stress over Crop Health and Intervention by Various Biostimulants. J. Agric. Food Chem..

[144] Sarraf M., Vishwakarma K., Kumar V., Arif N., Das S., Johnson R., Janeeshma E., Puthur J.T., Aliniaeifard S., Chauhan D.K. (2022). Metal/Metalloid-Based Nanomaterials for Plant Abiotic Stress Tolerance: An Overview of the Mechanisms. Plants.

[145] Zaman W., Ayaz A., Park S. (2025). Nanomaterials in Agriculture: A Pathway to Enhanced Plant Growth and Abiotic Stress Resistance. Plants.

[146] Chen L., Huang F., Liu J., Yang R., Hu Q., Li T., Zeng Y., Dai W., Qiu T., White J.C. (2025). Engineered Nanomaterials Enhance Crop Drought Resistance for Sustainable Agriculture. J. Agric. Food Chem..

[147] Zhao L., Wang W., Fu X., Liu A., Cao J., Liu J. (2022). Graphene Oxide, a Novel Nanomaterial as Soil Water Retention Agent, Dramatically Enhances Drought Stress Tolerance in Soybean Plants. Front. Plant Sci..

[148] Feng Y., Zhang Z., Ding Z., Chen K., Zhou Z., Ren X., Liang J., Huang Y., Shah A.A., Li Y. (2025). Enhanced Photosynthesis in *Zea maize* with the Biofortification of ZIF-8 Electron Bridge to Mediate Growth Improvement under Drought. Chem. Eng. J..

[149] Li Y., Tao Y., Xu W., Wu H., Li G., Yue L., Gu J., Li F., Wu H., Giraldo J.P. (2025). Mn_3_O_4_ Nanoparticles Maintain ROS Homeostasis to Modulate Stomatal Aperture to Improve Cotton Drought Tolerance. Environ. Sci. Nano.

[150] Sidhu A.K., Sharma M., Bhickchand Agrawal S., Pradip Bhavsar P., Samota M.K. (2024). Nanomaterial Strategies for Enhancing Plant Resilience in the Face of Temperature Stress. CABI Agric. Biosci..

[151] Halaji B., Haghighi M., Amiri A., Kappel N. (2023). Effects of Potassium and Nanocapsule of Potassium on Pepper Growth and Physiological Changes in High-Temperature Stress. J. Soil Sci. Plant Nutr..

[152] Song Y., Jiang M., Zhang H., Li R. (2021). Zinc Oxide Nanoparticles Alleviate Chilling Stress in Rice (*Oryza sativa* L.) by Regulating Antioxidative System and Chilling Response Transcription Factors. Molecules.

[153] Fu C., Khan M.N., Yan J., Hong X., Zhao F., Chen L., Ma H., Li Y., Li J., Wu H. (2023). Mechanisms of Nanomaterials for Improving Plant Salt Tolerance. Crop Environ..

[154] Zhu J.-K. (2016). Abiotic Stress Signaling and Responses in Plants. Cell.

[155] Li Z., Kekeli M.A., Jiang Y., Rui Y. (2025). Progress and Prospect of Saline-Alkaline Soil Management Technology: A Review. Appl. Sci..

[156] Cheng X. (2025). Tannic Acid-Iron Nanomaterial Enhances Rice Growth and Antioxidant Defense under Salt Stress. Front. Plant Sci..

[157] Li Y., Xu R., Ma C., Yu J., Lei S., Han Q., Wang H. (2023). Potential Functions of Engineered Nanomaterials in Cadmium Remediation in Soil-Plant System: A Review. Environ. Pollut..

[158] Zhou P., Adeel M., Shakoor N., Guo M., Hao Y., Azeem I., Li M., Liu M., Rui Y. (2020). Application of Nanoparticles Alleviates Heavy Metals Stress and Promotes Plant Growth: An Overview. Nanomaterials.

[159] Cao X., Chen X., Liu E., Wang C., Li X., Yue L., White J.C., Wang Z., Xing B. (2025). Metalloid Nanomaterials Alleviate Arsenic Phytotoxicity and Grain Accumulation in Rice: Mechanisms of Abiotic Stress Tolerance and Rhizosphere Behavior. Environ. Sci. Technol..

[160] Yan J., Kong N., Liu Q., Wang M., Lv K., Zeng H., Chen W., Luo J., Lou H., Song L. (2023). Ti_3_C_2_Tx MXene Nanosheets Enhance the Tolerance of Torreya Grandis to Pb Stress. J. Hazard. Mater..

[161] Panahirad S., Dadpour M., Gohari G., Akbari A., Mahdavinia G., Jafari H., Kulak M., Alcázar R., Fotopoulos V. (2023). Putrescine-Functionalized Carbon Quantum Dot (Put-CQD) Nanoparticle: A Promising Stress-Protecting Agent against Cadmium Stress in Grapevine (*Vitis vinifera* Cv. *Sultana*). Plant Physiol. Biochem..

[162] Moustafa-Farag M., Almoneafy A., Mahmoud A., Elkelish A., Arnao M., Li L., Ai S. (2019). Melatonin and Its Protective Role against Biotic Stress Impacts on Plants. Biomolecules.

[163] Saberi Riseh R., Hassanisaadi M., Vatankhah M., Soroush F., Varma R.S. (2022). Nano/Microencapsulation of Plant Biocontrol Agents by Chitosan, Alginate, and Other Important Biopolymers as a Novel Strategy for Alleviating Plant Biotic Stresses. Int. J. Biol. Macromol..

[164] Ma C., Borgatta J., Hudson B.G., Tamijani A.A., De La Torre-Roche R., Zuverza-Mena N., Shen Y., Elmer W., Xing B., Mason S.E. (2020). Advanced Material Modulation of Nutritional and Phytohormone Status Alleviates Damage from Soybean Sudden Death Syndrome. Nat. Nanotechnol..

[165] González-García Y., Cadenas-Pliego G., Alpuche-Solís Á.G., Cabrera R.I., Juárez-Maldonado A. (2022). Effect of Carbon-Based Nanomaterials on Fusarium Wilt in Tomato. Sci. Hortic..

[166] Borgatta J., Shen Y., Tamez C., Green C., Hedlund Orbeck J.K., Cahill M.S., Protter C., Deng C., Wang Y., Elmer W. (2023). Influence of CuO Nanoparticle Aspect Ratio and Surface Charge on Disease Suppression in Tomato (*Solanum lycopersicum*). J. Agric. Food Chem..

[167] Yu H., Zhang S., Zhang X., Gao L., Chi W., Zhu M., Yuan Y., Zhang Y. (2025). Novel ZnO-TiO_2_@MSC Nanomaterial Based on Corn Stover Template Enhances Disease Resistance in Tomato Plants. J. Environ. Manag..

[168] Narware J., Singh S.P., Chakma J., Ranjan P., Behera L., Das P., Manzar N., Kashyap A.S. (2024). Enhancing Tomato Growth and Early Blight Disease Resistance through Green-Synthesized Silver Nanoparticles: Insights into Plant Physiology. S. Afr. J. Bot..

[169] Ejaz M., Raja N.I., Khan S.A., Mashwani Z.-U.-R., Hanif A., Iqbal M., Hussain M., Syed A., Iqbal R.K., Qureshi H. (2023). Biosynthesized Silver Nanoparticles Ameliorate Biotic Stress in Rice (*Oryza sativa*) by Intricating Biochemical and Mineral Profile. Pak. J. Bot..

[170] Cao X., Chen X., Liu Y., Wang C., Yue L., Elmer W.H., White J.C., Wang Z., Xing B. (2023). Lanthanum Silicate Nanomaterials Enhance Sheath Blight Resistance in Rice: Mechanisms of Action and Soil Health Evaluation. ACS Nano.

[171] Xiao Z., Ji H., Yue L., Chen F., Yan X.-P., Wang Z., Rasmann S. (2024). Nano-Chitosan Boosts Sesame Plant Anti-Herbivore Defenses and Seed Nutritional Metabolites. Environ. Sci. Nano.

[172] Halder K., Chaudhuri A., Abdin M.Z., Majee M., Datta A. (2022). RNA Interference for Improving Disease Resistance in Plants and Its Relevance in This Clustered Regularly Interspaced Short Palindromic Repeats-Dominated Era in Terms of dsRNA-Based Biopesticides. Front. Plant Sci..

[173] El-Saadony M.T., Saad A.M., Najjar A.A., Alzahrani S.O., Alkhatib F.M., Shafi M.E., Selem E., Desoky E.-S.M., Fouda S.E.E., El-Tahan A.M. (2021). The Use of Biological Selenium Nanoparticles to Suppress *Triticum aestivum* L. Crown and Root Rot Diseases Induced by *Fusarium* Species and Improve Yield under Drought and Heat Stress. Saudi J. Biol. Sci..

[174] Wang Y., Welch Z.S., Ramirez A.R., Bouchard D.C., Schimel J.P., Gardea-Torresdey J.L., Holden P.A. (2019). Effects of Carbonaceous Nanomaterials on Soil-Grown Soybeans under Combined Heat and Insect Stresses. Environ. Chem..

[175] Singh A.V., Shelar A., Rai M., Laux P., Thakur M., Dosnkyi I., Santomauro G., Singh A.K., Luch A., Patil R. (2024). Harmonization Risks and Rewards: Nano-QSAR for Agricultural Nanomaterials. J. Agric. Food Chem..

[176] Zafar H., Javed R., Zia M. (2023). Nanotoxicity Assessment in Plants: An Updated Overview. Environ. Sci. Pollut. Res..

[177] Zhang T., Wang Q., Rui Y. (2025). The Impact of Nanomaterials on Plant Health: A Review of Exposure, Toxicity, and Control. Environ. Sci. Nano.

[178] Mutalik C., Nivedita, Sneka C., Krisnawati D.I., Yougbaré S., Hsu C.-C., Kuo T.-R. (2024). Zebrafish Insights into Nanomaterial Toxicity: A Focused Exploration on Metallic, Metal Oxide, Semiconductor, and Mixed-Metal Nanoparticles. Int. J. Mol. Sci..

[179] Azizah R.N., Verheyen G.R., Shkedy Z., Van Miert S. (2024). Testing for In Vitro Genetic Toxicity in High Dimensional Nanomaterial Dose-Response Experiments. J. Nanoparticle Res..

[180] Makhado B.P., Oladipo A.O., Gumbi N.N., De Kock L.A., Andraos C., Gulumian M., Nxumalo E.N. (2024). Unravelling the Toxicity of Carbon Nanomaterials–from Cellular Interactions to Mechanistic Understanding. Toxicol. In Vitro.

[181] Xie J., Wu Y., Chen Z., Zheng M., Yang Q., Mo M., Liu J., Chen L. (2025). Ocular Toxicity and Potential Mechanism of Nanomaterials: An Issue Worthy of Investigation. Ecotoxicol. Environ. Saf..

[182] Sree S.S., Al-zharani M., Nasr F.A., Alneghery L.M., Kumar T.T.A., Sureshkumar B.T., Mohamed J.M.M., Ravichandran M., Dineshkumar R. (2025). Innovative Bio-Amelioration Strategies for Sustainable Reclamation of Salt-Affected Agroecosystems: A Review. Plant Soil.

[183] Rodríguez-Seijo A., Santás-Miguel V., Arenas-Lago D., Arias-Estévez M., Pérez-Rodríguez P. (2025). Use of Nanotechnology for Safe Agriculture and Food Production: Challenges and Limitations. Pedosphere.

[184] Thiruvengadam M., Chi H.-Y., Choi H.-J., Jung B.-S., Lee S.-B., Park Y., Jeon D., Ciftci F., Shariati M.A., Kim S.-H. (2025). Sustainable and Smart Nano-Biosensors: Integrated Solutions for Healthcare, Environmental Monitoring, Agriculture, and Food Safety. Ind. Crops Prod..

[185] Rani N., Sagar N.A., Chauhan A., Mondal A. (2025). Green Synthesis of ZnO Nanoparticles: Characterization and Emerging Applications in Sustainable Agriculture. Ind. Crops Prod..

[186] Sharma V., Thakur S. (2025). Green Nano-Pesticides from Plant Sources: Synthesis, Mechanisms, Environmental Impacts, and Prospects for Sustainable Agriculture. Pestic. Biochem. Physiol..

[187] Singh P., Gupta P., Verma V., Yadav N., Pandey N.K., George S.C., Tawiah B. (2024). Nanotechnology: Social Acceptance, Cultural Impact and Privacy. Nanotechnology in Societal Development.

